# Screening the active compounds of *Phellodendri Amurensis* cortex for treating prostate cancer by high-throughput chinmedomics

**DOI:** 10.1038/srep46234

**Published:** 2017-04-06

**Authors:** Xian-Na Li, Aihua Zhang, Meijia Wang, Hui Sun, Zhidong Liu, Shi Qiu, Tianlei Zhang, Xijun Wang

**Affiliations:** 1 Sino-America Chinmedomics Technology Collaboration Center, National TCM Key Laboratory of Serum Pharmacochemistry, Chinmedomics Research Center of TCM State Administration, Metabolomics Laboratory, Department of Pharmaceutical Analysis, Heilongjiang University of Chinese Medicine, Heping Road 24, Harbin 150040, China

## Abstract

Screening the active compounds of herbal medicines is of importance to modern drug discovery. In this work, an integrative strategy was established to discover the effective compounds and their therapeutic targets using *Phellodendri Amurensis* cortex (PAC) aimed at inhibiting prostate cancer as a case study. We found that PAC could be inhibited the growth of xenograft tumours of prostate cancer. Global constituents and serum metabolites were analysed by UPLC-MS based on the established chinmedomics analysis method, a total of 54 peaks in the spectrum of PAC were characterised *in vitro* and 38 peaks were characterised *in vivo*. Among the 38 compounds characterised *in vivo*, 29 prototype components were absorbed in serum and nine metabolites were identified *in vivo*. Thirty-four metabolic biomarkers were related to prostate cancer, and PAC could observably reverse these metabolic biomarkers to their normal level and regulate the disturbed metabolic profile to a healthy state. A chinmedomics approach showed that ten absorbed constituents, as effective compounds, were associated with the therapeutic effect of PAC. In combination with bioactivity assays, the action targets were also predicted and discovered. As an illustrative case study, the strategy was successfully applied to high-throughput screening of active compounds from herbal medicine.

Prostate cancer is one of the most common malignant tumours of the urogenital system in elderly males, especially in European and American men^1^,^2^. With the rapid increase of its incidence and mortality rate, more and more patients are suffering from the associated emotional and physical burden, and the mortality rate of prostate cancer is second only to lung cancer^3^,^4^: because of the high progression, invasion, and metastasis rates of prostate cancer, it causes the highest mortality rate and has the lowest survival rate^5^,^6^,^7^. Prostate cancer threatens male health at all times^8^. In this context, most research focuses on the prevention and treatment of prostate cancer, including discovery of natural herbs for prostate cancers treatment^9^,^10^,^11^.

*Phellodendri Amurensis* cortex (PAC), a well-known herbal medicine in China, has been widely used for immunosuppression, antipyretic, anti-inflammatory, antibacterial, anticancer, anti-ulcer, antioxidant, anti-gout, reduction of blood pressure activities, and many other pharmacological activities^12^,^13^,^14^. It was widely applied in clinics for cancer, pneumonia, tuberculosis hepatocirrhosis, gout, dysentery, eczema, conjunctivitis, osteoarthritic cartilage, and so on^15^,^16^. The main chemical components of PAC are: alkaloids, limonin, flavones, terpenes, sterols, phenolic acid, and others, such as berberine, tetrahydroberberine, phellodendrine, magnoflorine, jatrorrhizine, palmatine, obacunone, limonin, obacunonic, hyperin, phellozid, β-sitosterol, and dictamnoilde^17^,^18^,^19^,^20^. Recent research shows that, PAC has a therapeutic effect in prostate cancer, but the previous research focused on the chemical composition analysis and pharmacodynamicals, this work addresses the active components and action mechanism of PAC against prostate cancer^21^,^22^,^23^,^24^,^25^,^26^.

Recently, “omic” technologies have become the important methods in biological research. As an important part of systemic biology, metabolomics is a front subject in the field of medical research^27^,^28^,^29^. It can represent the pathophysiological changes of integrated living systems which are caused by endogenous or exogenous factors^30^,^31^,^32^. Because the chemical compositions of herbal medicine are complicated, scientific research could not always reveal the effective constituents and the mechanisms of action of traditional medicine. Metabolomics, through analysing the dynamic changes of endogenous metabolites *in vivo* after oral administration of herbal medicine, tries to determine the therapeutic target and therapeutic receptor of herbal medicines used in the treatment of disease^33^,^34^,^35^,^36^,^37^,^38^,^39^. We have established a new theory called chinmedomics, combining serum pharmacochemistry and metabolomics, it can reveal the links between the chemical constituents in serum exogenous and endogenous metabolic biomarkers, and then to discover the chemical constituents best correlated with the endogenous metabolic biomarkers as effective material bases for herbal medicine^40^,^41^. This study examined the mechanism of action of PAC against prostate cancer through use of a chinmedomics strategy.

## Results

### *In vivo* tumourigenicity and dug response

**Figure 1:**
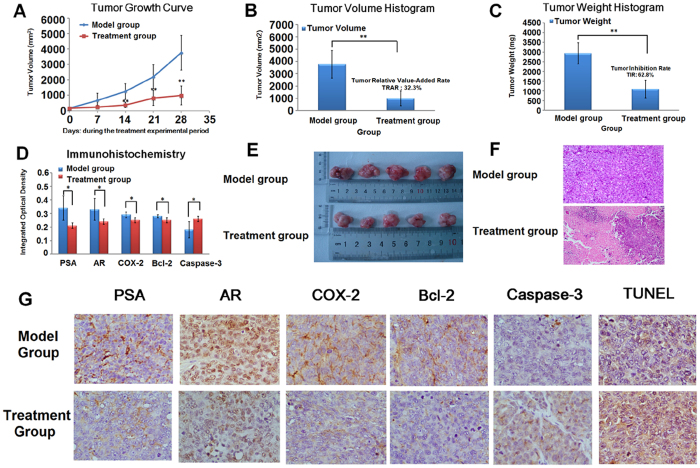
Comparisons of tumour growth, histopathology, immunohistochemical, and TUNEL analyses between model, and treatment, groups. (**A**) The average tumour volume among model, and treatment, group samples during the treatment experimental period. (**B**) Comparison of average tumour volume between model, and treatment, groups over 28 days. (**C**) Comparisons of average tumour mass between model, and treatment, groups for 28 days. (**D**) Comparisons of integrated optical density (IOD) value of PSA, AR, COX-2, Bcl-2, and Caspase-3 between model, and treatment, groups. (**E**) Representative tumour among model, and treatment, groups. (**F**) H&E staining for histological evaluation (magnification 100x). (**G**) TUNEL analysis and the expression of PSA, AR, COX-2, Bcl-2, and Caspase-3 in immunohistochemical analysis (magnification 400x). *P < 0.05; **P < 0.01 *v*. model group.

We investigated the therapeutic efficiency of PAC in inhibiting the growth of xenograft tumours after the subcutaneous inoculation of 22RV1 human prostate cancer cells into male BALB/c-nude mice. Results showed that, both mice had palpable tumour nodules at 15 days post-injection with a tumour formation rate of 100%. The xenograft tumours grew quickly after tumour nodule formation and nearly 21 days post-injection the average tumour volume reached 100 to 300 mm^3^. At the beginning of xenograft tumour growth, the weight of the mice was unchanged: following the growth of xenograft tumours, their activity and weight had slightly decreased. During the experimental period, both mice were healthy. PAC therapy was started as the average tumour volume reached 100 to 300 mm^3^. Following treatment, the tumour volume in the treatment group was significantly smaller than in the model group. On day 14, the tumour volume in the treatment group showed a statistically significant difference compared to the model group (*P* < 0.01). At the end of the trial, the tumour volume in the treatment group was 988.57 ± 606.23 mm^3^ and in the model group it was 3753.65 ± 1119.27 mm^3^ (*P* < 0.01). The relative tumour volume in treatment, and model, groups were 8.20 ± 5.49 mm^3^ and 23.36 ± 8.15 mm^3^, respectively (*P* < 0.01). The tumour relative size increase was 32.3%. After the mice were sacrificed, we harvested and weighed the tumours. The tumour weights in treatment, and model, groups were 1089.8 ± 455.2 mg and 2932.8 ± 544.2 mg, respectively (*P* < 0.01), the tumour inhibition rate was 62.8%. The result of tumour volume, relative tumour volume, tumour weight, tumour relative value-added rate and inhibition rate are shown in Fig. 1A–C, and Supplemental Table 1. Result indicates that PAC could dramatically delay the growth of 22RV1 human prostate cancer cell-xenograft tumours.

### Histopathology, immunohistochemical, and TUNEL analysis

By using Image-Pro Plus (IPP) software to quantify the integrated optical density value (IOD) of COX-2, PAS, AR, Bcl-2, and Caspase-3, result indicated that the IOD optical density value of PSA, AR, COX-2, Bcl-2 staining in model group tissue samples were higher than the IOD numerical values in the treatment group (*P* < 0.05), while the IOD value of Caspase-3 staining in model group tissue samples was reduced compared with the treatment group (*P* < 0.05). The expression of COX-2, PAS, AR, Bcl-2, and Caspase-3 of the treatment, and model, groups are shown in Fig. 1D and G, and Supplemental Table 2. Results showed that the effect of PAC treatment on prostate cancer is mainly achieved through reducing the expression of AR and inhibiting the inflammatory microenvironment *in vivo*, finally inhibiting prostate cancer proliferation and inducing prostate cancer apoptosis.

### Tumour apoptosis in mice

TUNEL staining was used to calculate the cell apoptosis rate to see if PAC inhibited prostate cancer tumour growth. Result shows that, after TUNEL staining, the apoptotic positive cells were coloured by tan or brown, and the normal tumour cells were coloured blue (Fig. 1G). From each group we selected five high-power fields to calculate the percentage rate of apoptotic cells for every 200 cells using the following formula: the cell apoptosis rate = number of positive apoptotic cell/ 200 × 100%. Finally, the cell apoptosis rates of the treatment, and model, groups were (52.9 ± 6.9)% and (19.2 ± 5.1)%, respectively. The number of positive cells was increased in tumour tissues after PAC treatment, thus the cell apoptosis rate of the treatment group was higher than those in the model group (*P* < 0.01). Result indicated that PAC could induce the apoptosis of prostate cancer cells and inhibit the growth of prostate cancer tumour.

### Metabolomic analysis of PAC and its effect on cancer

#### Multivariate statistical analysis of metabolite profiling

**Figure 2:**
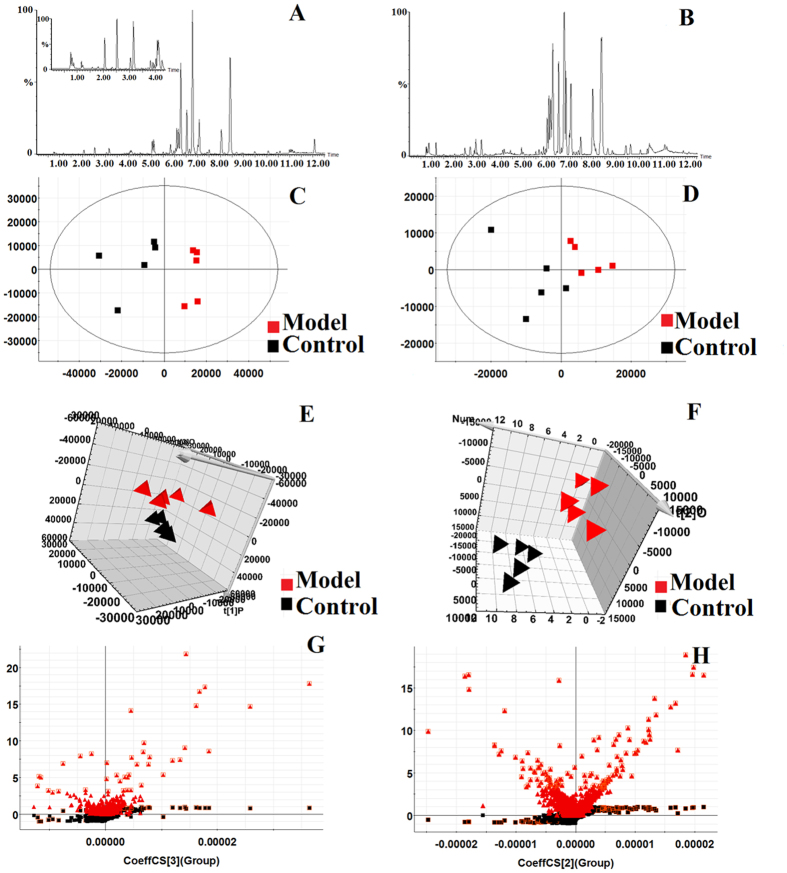
Metabolic profile characterisation and multivariate data analysis. (**A**) UPLC-G2-Si-MS/MS BPI serum chromatograms in positive mode; (**B**) UPLC-G2-Si-MS/MS BPI serum chromatograms in negative mode; (**C**) PCA score plots for control and model groups in positive mode; (**D**) PCA score plots for control and model groups in negative mode; (**E**) 3-d score plots of OPLS-DA based on serum metabolites discriminating between control and model groups in positive mode; (**F**) 3-d score plots of OPLS-DA based on serum metabolites discriminating between control and model groups in negative mode; (**G**) Metabolite biomarkers in the VIP & S-plot between control and model group in positive mode; (**H**) Metabolite biomarkers in the VIP & S-plot between control and model groups in negative mode.

The basic peak intensity (BPI) chromatograms of the serum samples in each group are shown in Fig. 2A and B. Progenesis QI software and EZinfo 2.0 software were used to explore the metabolite biomarkers, which play a key role in the change of the metabolic profile. Firstly, the raw data obtained from UPLC/MS were pre-processed by the Progenesis QI software, and then processed by EZinfo 2.0 software for multivariate data analyses, including PCA, PLS-DA, and OPLS-DA. The PCA result shows that there was a distinguished classification between the clustering of the control, and model, groups. PCA results indicated biochemical perturbation *in vivo* after injecting the 22RV1 human prostate cancer cells into model group mice. The VIP-plot and S-plot obtained from OPLS analysis were used to find the metabolite biomarkers of prostatic cancer. In the S-plot the further the metabolite ions from the origin, the higher the VIP value of the relative metabolite ion: the higher VIP value represents a greater contribution to the difference between groupsrol group, it can be confirmed as being part of the metabolite biomarkers of prostatic cancer. The PCA, 3D-plot, VIP-plot, and S-plot are shown in Fig. 2C–H.

#### Metabolite identification

**Figure 3:**
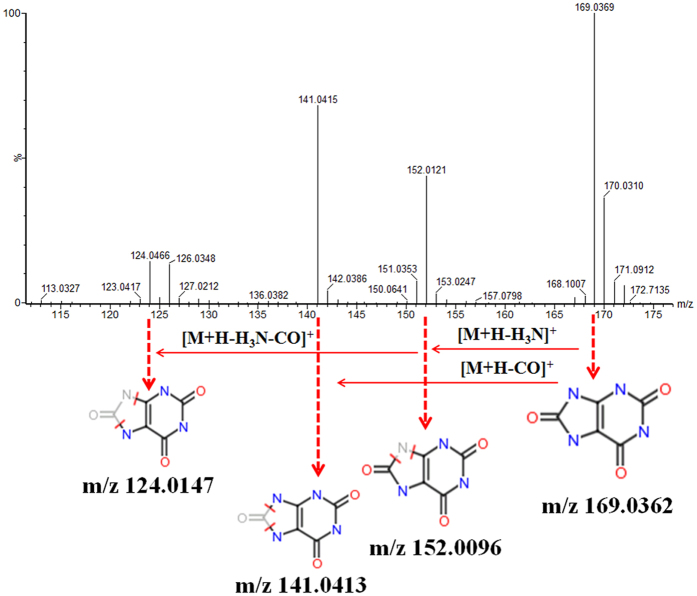
Chemical structure and mass fragment information: uric acid in positive mode.

**Figure 4:**
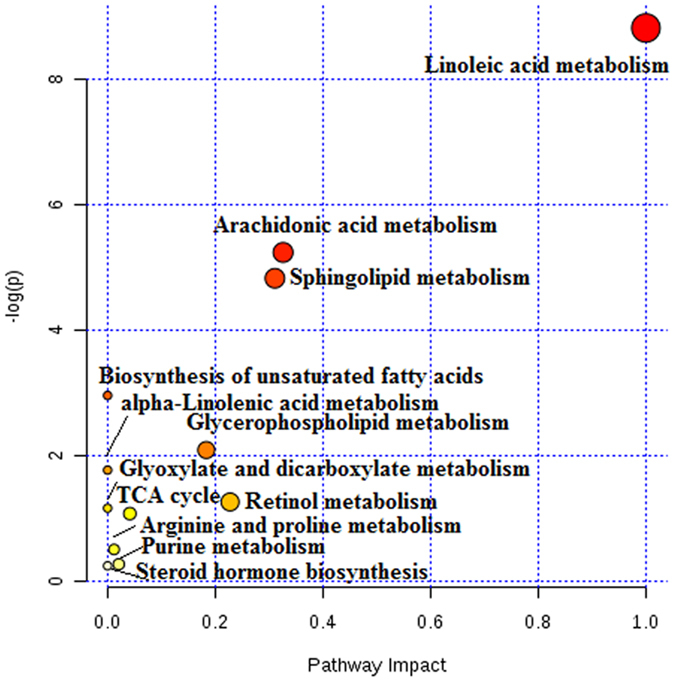
Pathway analysis with the MetaboAnalyst tool.

**Figure 5:**
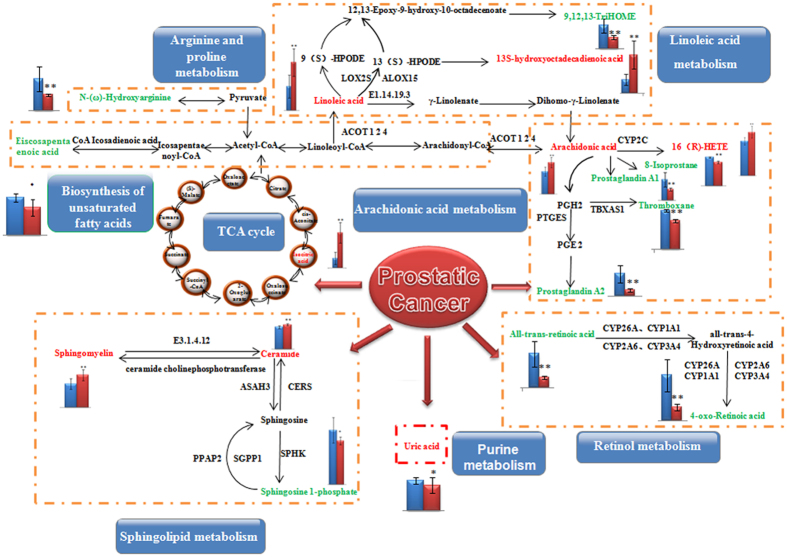
Construction of the altered metabolic network associated prostate cancer model based on KEGG pathway database.

The identification of the metabolite biomarkers can be conducted using retention time data, precise MS and MS/MS information, and these were detected using the established UPLC-G2-Si-MS/MS method. We characterised the structure of metabolite biomarkers and analysed the related metabolic pathways based on previous benchmarks. Accordingly, a total of 34 metabolite biomarkers (VIP > 3.0, *P* < 0.05) were identified (Table 3). For example, the identification process was as follows: ESI positive ion mode gave an [M+H]^+^ ion at m/z 169.0365 and Rt at 0.86 min with high VIP values; based on the MS and MS/MS data, the molecular formula of the ion was speculated as being C_5_H_4_N_4_O_3_, the MS/MS screening were: m/z 169 [M+H]^+^; 152 [M+H-H_3_N]^+^; 141 [M+H-CO]^+^; 124 [M+H-CH_3_NO]^+^ which was characterised as uric acid. The mass spectrum and proposed fragmentation pathway of uric acid are showed in Fig. 3. Among the 34 metabolite biomarkers, 17 biomarkers were up-regulated and 17 down-regulated in the model group. The MetaboAnalyst platform revealed the metabolic pathways which were associated with prostate cancer. Result indicates that 12 metabolism pathways were associated with the pathological process of prostate cancer, such as linoleic acid metabolism, arachidonic acid metabolism, sphingolipid metabolism, glycerophospholipid metabolism, alpha-linolenic acid metabolism, retinol metabolism, glyoxylate and dicarboxylate metabolism, arginine and proline metabolism, purine metabolism, citrate cycle (TCA cycle), biosynthesis of unsaturated fatty acids, and steroid hormone biosynthesis. The detailed metabolic pathways analysis result is shown in Fig. 4 and Supplemental Table 4: according to analysis the correlation between metabolite biomarkers and metabolic pathways, the metabolic networks of metabolite biomarkers were described (Fig. 5).

#### Protective effects of PAC against cancer

**Figure 6:**
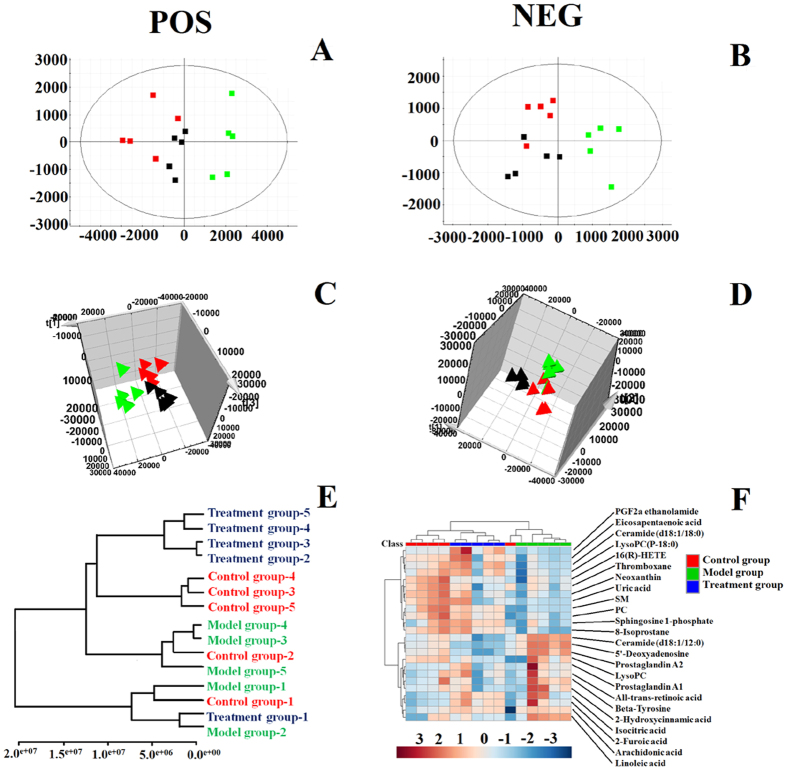
Metabolic profile characterisation and multivariate data analysis. (**A**) PCA score plots for control, model, and treatment groups in positive mode; (**B**) PCA score plots for control, model, and treatment groups in negative mode; (**C**) 3-d score plots of OPLS-DA based on serum metabolites discriminating between control, model, and treatment groups in positive mode; (**D**) 3-d score plots of OPLS-DA based on serum metabolites discriminating between control, model, and treatment groups in negative mode; (**E**) Dendrogram visualisation for serum samples from the control, model, and treatment groups; (**F**) Heatmap visualisation for serum samples from the control, model, and treatment groups.

**Figure 7:**
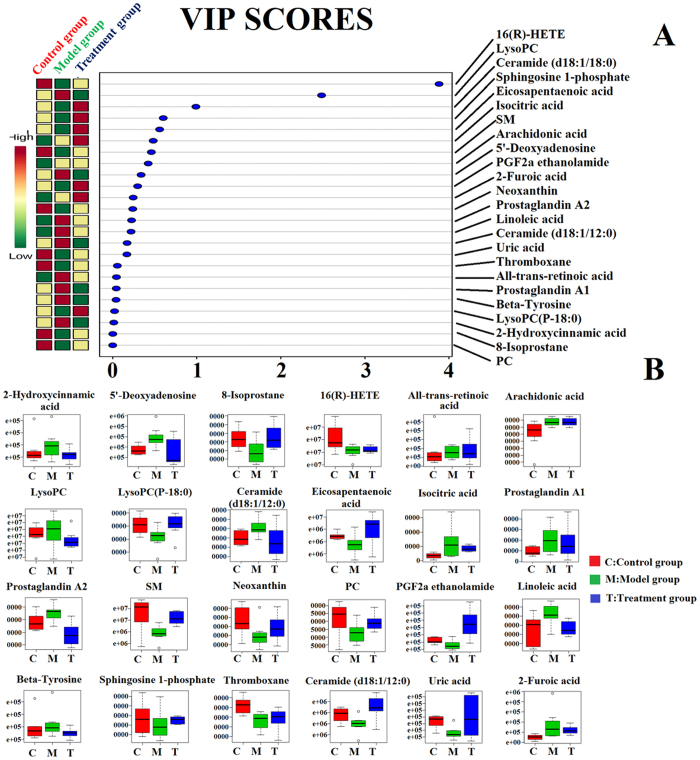
Significant changes in metabolite biomarker candidates in control, model, and treatment groups. (**A**) VIP scores of the metabolite marker candidates. (**B**) Relative signal intensities of the metabolites identified by UPLC-G2-Si-MS/MS.

Principal component analysis (PCA) revealed the metabolic profile of the control group, model group, and treatment group, and provided a score plot diagram which can reflect the degree of separation among the different groups. Results showed that there was a clear separation between the control group, model group and treatment group and the metabolic profile of the treatment group was closer to the control group than the model group (Fig. 6A–D). The MetaboAnalyst system revealed the effects of PAC against prostate cancer. Hierarchical cluster analysis showed that the metabolic profile in the treatment group was close to that of the control group, it was indicated that PAC had therapeutic efficacy, the dendrogram and heatmap are shown in Fig. 6E and F. Through analysis of the VIP scores of biomarkers in different groups, it was shown that the relative concentration of these metabolites could be reversed after taking PAC, the VIP scores are shown in Fig. 7A. The result indicated that PAC could return the metabolic profile to its normal state (Fig. 7B). PAC could completely reverse 24 biomarkers to abnormal levels, including 16(R)-HETE, LysoPC, SM, Eicosapentaenoic acid, Ceramide (d18:1/12:0), Sphingosine 1-phosphate, LysoPC(P-18:0), Arachidonic acid, 8-Isoprostane, Linoleic acid, Prostaglandin A1, Neoxanthin, Ceramide (d18:1/18:0), Uric acid, Isocitric acid, 5′-Deoxyadenosine, PGF2a ethanolamide, Beta-Tyrosine, 2-Furoic acid, Thromboxane, 2-Hydroxycinnamic acid, All-trans-retinoic acid, Prostaglandin A2, and PC, these metabolite biomarkers were involved in six metabolic pathways. Results indicated that PAC displayed an obvious effect in the treatment of prostate cancer through adjusting the disturbed metabolic pathways such as purine metabolism, citrate cycle (TCA cycle), arachidonic acid metabolism, retinol metabolism, sphingolipid metabolism, glycerophospholipid metabolism, and many other metabolic pathways.

### Constituent analysis of PAC

**Figure 8:**
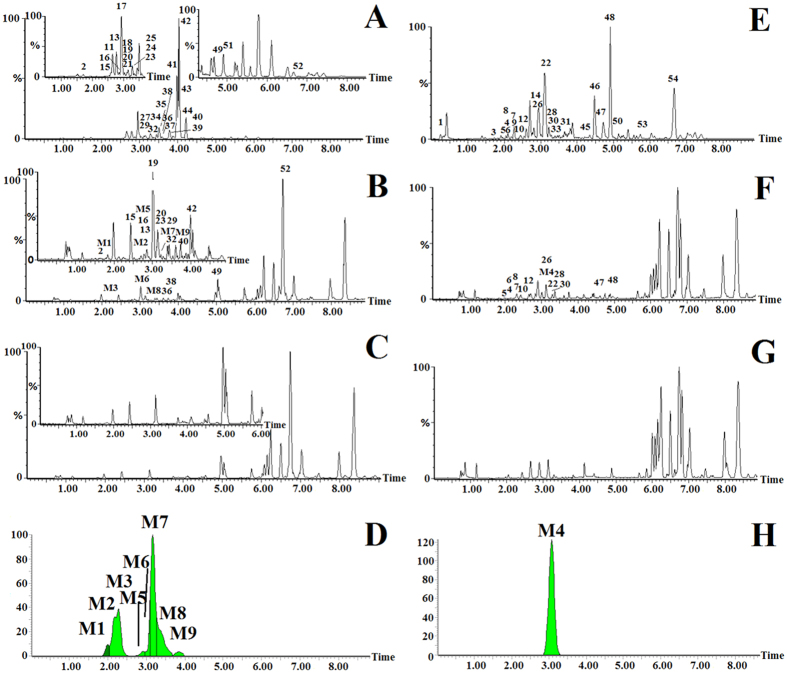
ESI base peak ion (BPI) chromatogram of the PAC as analysed by UPLC-G2-Si-MS/MS. Positive ion mode (**A**), PAC extracts; (**B**), PAC serum; (**C**), control serum; (**D**), extracted ion chromatograms of metabolites with MetaboLynx tool; Negative ion mode (**E**), PAC extracts; (**F**), PAC serum; (**G**), control serum; (**H**), extracted ion chromatograms of metabolites with MetaboLynx tool. (The constituents in PAC extracts were marked with a number and the metabolites in the drug serum were marked M: both peak numbers are listed in Supplementary Tables 5 and 6).

UPLC-G2-Si-MS/MS and MetaboLynx^TM^ software were used to analyse and characterise the chemical components of PAC. The structure of chemical components can be characterised by retention time, and MS/MS information. In positive and negative ion mode, against benchmarks we analysed the high- and low-energy data about each chemical component. Finally, we characterised 54 chemical components of PAC *in vitro*, as consisting of 30 compounds in positive ion mode and 24 compounds in negative ion mode. The ESI BPI chromatogram of the PAC by UPLC-G2-Si-MS is shown in Fig. 8A and E and the structures and the mass data of ESI^+^ and ESI^−^ MS spectra of the identified compounds are shown in Supplemental Table 5.

### *In vivo* analysis of PAC using Metabolynx^TM^

Metabolynx software and multiple pattern recognition methods were used to compare the differences of raw data between the dosed group and the control group, and then we screened the absorbed constituents and metabolites of PAC *in vivo*. Only when the ion peaks were present in the analyte chromatogram and absent in the control chromatogram, did we consider it a prototype peak or a metabolite peak. The constituent of each prototype was identified by comparing the MS and MS/MS data with full scan analysis the chemical compounds of PAC sample *in vitro*: the metabolite screening of PAC was processed by the MetaboLynx program and this indicated ethylene loss, hydroxymethylene loss, demethylation, 2× hydroxylation, and glucuronide conjugation as the predominant metabolic pathways of PAC. The extracted ion chromatogram of plasma samples with MetaboLynx tool are shown in Fig. 8D and H. Finally, we characterised 38 chemical components of PAC *in vivo*, consisting of 29 prototype compounds and nine metabolites of PAC absorbed in *in vivo* ion mode. The ESI BPI chromatogram of the PAC by UPLC-G2-Si-MS is shown in Fig. 8 and all compound information is shown in Supplemental Table 6.

### Correlation analysis between marker metabolites and absorbed constituents

**Figure 9:**
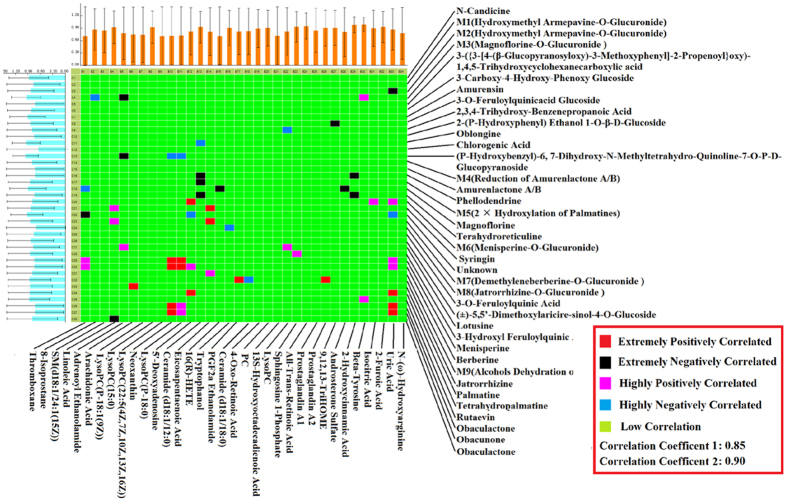
PCMS analysis between metabolic biomarkers and chemical constituents of PAC.

Chinmedomics provided a way of discovering the active compounds of PAC absorbed *in vivo*. Through establishing a correlation model between the metabolic biomarkers and the chemical compounds, the relevant parameters were: 0.9 < |r| ≤ 1 means extremely high correlation and 0.85 < |r| ≤ 0.9 means a high correlation, and then the active compounds contributed to the main therapeutic effect of PAC in the treatment of prostate cancer can be discovered. The heatmap of the correlation between metabolic biomarkers and chemical compounds of PAC absorbed *in vivo* are shown in Fig. 9. Results shows that 10 chemical compounds were significantly correlated with metabolite biomarkers of prostate cancer, they were determined as being potential effective material bases of PAC against prostatic cancer. The chemical compounds NO of C4 (M3: Magnoflorine-O-Glucuronide), C13 ((p-hydroxybenzyl)-6, 7-dihydroxy-N-methyltetrahydro iso-quinoline-7-O-p- D-glucopyranosid), C18 (Magnoflorine), C20 (M6: Menisperine-O-Glucuronide), C22 (unknown), C29 (Menisperine), C30 (Berberine), C32 (Jatrorrhizine), C36 (Obaculactone), C37 (Obacunone) had an extremely high correlation with the therapeutic effect of PAC against prostate cancer. The research results provide data revealing the potential effective constituents of PAC against prostate cancer: these potential effective constituents were mostly correlated with the metabolite biomarkers of thromboxane, 8-isoprostane, adrenoyl ethanolamide, neoxanthin, lysoPC, 16(R)-HETE, prostaglandin A2, androsterone sulfate, uric acid, isocitric acid, *etc*. PAC mainly affected seven metabolism pathways to treat prostate cancer, including arachidonic acid metabolism, retinol metabolism, sphingolipid metabolism, linoleic acid metabolism, glycerophospholipid metabolism, arginine and proline metabolism, and the citrate cycle (TCA cycle).

### Therapeutic target prediction of the correlated ingredients

Network pharmacology was used to investigate the protein target relative to its active constituent thus discovering the mechanism of action on the therapeutic target. Ten compounds absorbed into the blood were highly correlated with the therapeutic effect of PAC in prostate cancer, and these were selected to predict the biological targets. Then these 10 compounds were imported into the High-Throughput Docking database (http://www.cbligand.org/HTDocking/) and PharmMapper database (http://lilab.ecust.edu.cn/pharmmapper/index.php) to predict the targets. Results showed that eight protein targets (fitness score ranking in the top 20) were found (Supplemental Table 7), such as: androgen receptor, glucocorticoid receptor, vascular endothelial growth factor receptor 2, medium-chain specific acyl-CoA dehydrogenase, aspartate aminotransferase, fatty acid-binding protein, retinol-binding protein, and rhodopsin. These targets were put into the KEGG pathway database, results show that they were correlated with five pathways including regulation of lipolysis in adipocytes, lipid transport and metabolism, amino acid transport and metabolism, retinol metabolism, and prostate cancer metabolism.

## Discussion

Metabolomics is a powerful scientific technology, using a combination of an advanced analysis technology platform and the multivariate statistical analysis used to research changes in low molecular-weight metabolites and their relative metabolic pathway *in vivo*^42^. The serum pharmacochemistry of traditional Chinese medicine can provide an effective method with which to analyse the chemical constitutions absorbed into the blood after oral administration of traditional Chinese medicine^43^. Not all of the absorbed chemical constitutions were effective constituents of traditional Chinese medicine against disease. A chinmedomics research strategy can assess the correlation between metabolomics and serum pharmacochemistry by calculating the correlation coefficient of each chemical constituent and metabolic biomarker, and allow assessment of the efficacy of traditional Chinese medicine^44^.

In this present work, we established a blood metabolomics method based on UPLC-G2-Si-HDMS and combined this with the observation of xenograft tumour growth and its histopathology: comprehensive analysis of the changes in metabolomic profiling in prostate cancer model mice and evaluate the therapeutic effect of PAC against prostate cancer. The BALB/C-nude mouse model for prostate cancer was established by the use of right forelimb injection of 22RV1 human prostate cancer cells, and its reliability was tested by histopathological, and immunohistochemical analyses. This research evaluated the therapeutic effect and discovered the efficacy of a material based on PAC for treatment of prostate cancer. Histopathology results showed large tracts of cancer cell apoptosis in the treatment group. A TUNEL assay showed the same result: the numbers of positive cells were increased in xenograft tumour tissues after PAC treatment. Furthermore, we analysed PSA, AR, COX-2, Bcl-2, and caspase3 levels through immunohistochemical analysis: PAC could reduce the expression of PSA, AR, COX-2, and Bcl-2, and increase the expression of Caspase-3 in tumour samples. PSA is the specific biomarker of prostate cancer, the abnormal increases of PSA indicate the occurrence of prostate cancer^45^. AR plays an important role in the carcinogenesis and progress of prostate cancer; increased levels of AR expression predict the metastatic progression of prostate cancer^46^,^47^. Bcl-2 was the upstream protein of caspase3, and they are most relevant to cell apoptosis, and Caspase-3 is the major enzyme cleavage effector in the process of cell apoptosis and Bcl-2 is the main anti-apoptotic agent and the anti-oxidative protein is an inhibitor of cell apoptosis^48^. Both Caspase-3 and Bcl-2 play an important role in signal transduction of cell apoptosis^49^. COX-2 is an inflammatory mediator involved in the process of cell proliferation, migration, and apoptosis^50^,^51^. These results indicate that the effect of PAC treatment on prostate cancer is mainly achieved by adjusting the expression of PSA, AR, COX-2, Bcl-2, and caspase3, and by further inhibiting the inflammatory microenvironment *in vivo*, finally inhibiting prostate cancer cell proliferation and inducing prostate cancer cell apoptosis, offering further effective intervention in the growth of prostate cancer xenograft tumours. Based on blood metabolomics research, 34 prostate cancer metabolic biomarkers were discovered, and blood metabolomics showed that PAC could return the metabolic profile to its normal state and it reversed 24 metabolic biomarkers to abnormal levels, such as LysoPC, SM, Eicosapentaenoic acid, Sphingosine 1-phosphate, Arachidonic acid, Linoleic acid, Prostaglandin A1, Uric acid, Isocitric acid, All-trans-retinoic acid, Prostaglandin A2, PC, *etc*. PAC could reverse the abnormal expression of LysoPC, LysoPC(P-18:0), and PC: these metabolite biomarkers were involved in glycerophospholipid metabolism and choline metabolism. Choline metabolism is closely related to the occurrence and development of cancers^52^,^53^. PC plays an important role in maintaining the physiological activity of cell membranes, thus it was a potential target in cancer diagnosis^54^,^55^. Glycerophospholipid metabolism disorders are an important cancer marker: LysoPC, LysoPC(18:0) and sphingosine 1-phosphate are the signalling molecules of glycerophospholipid metabolism and the abnormal expression of these metabolite biomarkers indicates a disordered glycerophospholipid metabolism. Uric acid is involved in the purine metabolism pathway. It could promote cancer cell apoptosis, scavenging oxygen radicals, inhibit lipid peroxidation, and then as an antioxidant, prevent the deterioration of the tumour^56^,^57^. All-trans-retinoic acid is involved in retinol metabolism, it could regulate cell proliferation, differentiation, apoptosis, and immune response, and then play an important role in the growth of cancer cells^58^,^59^. Linoleic acid, as the main substance in arachidonic acid synthesis, is involved in linoleic acid metabolism. Arachidonic acid, Thromboxane, 16(R)-HETE, Prostaglandin A1, Prostaglandin A2, and 8-Isoprostane are involved in arachidonic acid metabolism. Arachidonic acid and its metabolites, as the main inflammatory substances, are taking part in the process of immune and inflammatory actions^60^,^61^. Thromboxane could promote cancer cell proliferation through enhancing the activity of cell mitosis^62^,^63^. Both linoleic acid metabolism and arachidonic acid metabolism are related to the malignant proliferation and metastasis of cancer cells. PAC could effectively reverse the abnormal expression of metabolite biomarkers. Through serum pharmacochemistry analysis, 38 compositions of PAC were found in serum including 29 prototype compounds and nine metabolites. Chinmedomics results show that 10 compositions were associated with the therapeutic effect of PAC against prostate cancer, and it may be the main efficacious material base of PAC, including Jatrorrhizine, Berberine, Obaculactone, Obacunone, Menisperine, p-hydroxybenzyl-6, Magnoflorine, an unknown compound, M3, M6, *etc*. It is closely related to eight protein targets and they were correlated with five pathways including regulation of lipolysis in adipocytes, lipid transport and metabolism, amino acid transport and metabolism, retinol metabolism, and prostate cancer metabolism. Chinmedomics provided data about material base of TCM, and the activity of these compounds was determined by further pharmacological research and then improved the accuracy of the conclusion. In this work, through research into the treatment effect of PAC against prostate cancer, results shows that the multiple components of PAC have obvious therapeutic effect against prostate cancer, and clarified its bioactive constituents and their mechanism of action.

Chinmedomics provides a way to clarify the efficacy of traditional Chinese medicine: it provides a bridge between the absorbed chemical constituents and metabolic biomarkers, and then through using an established mathematical model to calculate the correlation coefficient of each constituent, thus assessing the effective constituents of traditional Chinese medicine. Cluster analysis, correlation analysis, pattern recognition analysis, and so on, are used. In summary, chinmedomics may provide a way of evaluating the bioactive constituents of traditional Chinese medicine and then promote its modernisation.

### Experimental work

#### Chemicals and materials

Chemicals including HPLC grade acetonitrile were obtained from Merck (Darmstadt, Germany) and methanol was purchased from Fisher Scientific Corporation (Loughborough, UK). Distilled water was obtained from Watson’s Food & Beverage Co. Ltd (Guangzhou, China). Formic acid was obtained from Sigma-Aldrich (Mo, USA). Leucine enkephalin was purchased from Sigma-Aldrich (St. Louis, MO, USA). RPMI1640, TRIPSIN 0.25% (1×) were obtained from Hyclone (Logan, Utah, USA). Foetal bovine serum (FBS) was purchased from Gibco Laboratories (Grand Island, USA). Rabbit anti-Bcl-2 monoclonal antibody, Rabbit anti-COX-2 monoclonal antibody, Rabbit anti-AR monoclonal antibody, Mouse anti-PSA monoclonal antibody, Peroxidase-conjugated affini-pure goat anti-mouse IgG (H+L), Peroxidase-conjugated affini-pure goat anti-rabbit IgG (H+L), DAB kit, and *in situ* cell death detection kit POD was obtained from Roche Applied Science (Roche, Germany). 22RV1 human prostate cancer cells were obtained from Cobioer Biosciences Company (Nan Jing, China). PAC was purchased from Harbin Tongrentang Drug Store (Harbin, China) and authenticated by Prof. Xijun Wang, Department of Pharmacognosy of Heilongjiang University of Chinese Medicine.

#### Preparation of GHB samples

The PAC was ground into coarse powder samples (500 g each) and then extracted using reflux extraction methods with ten volumes of 75% methanol (twice), each time saw the reflux extracted for 1 h. We combine the three batches of filtrate and then freeze-dried them. Finally, we obtained 68.8 g of freeze-dried powder: 0.5% carboxymethyl cellulose solvent was used to dissolve the PAC freeze-dried powder at a concentration of 0.16 g•ml^−1^ and ultrasonicated for 30 min.

### Animal handing

Male BALB/c-nude mice (4–6-weeks-old) were obtained from the Laboratory Animal Centre of the Slac Laboratory Animal Company Ltd (Shanghai, China). Both of the BALB/c-nude mice were housed in an SFP room at 24 ± 2 °C and relative humidity of 50 ± 5%. A 12 h/12 h dark/light cycle was set and they were fed with standard laboratory water and food *ad libitum* for 7 days before the animal experiment started. Tumour cells (9 × 10^6^) were injected into the subcutis on the right forelimb of each BALB/c-nude mouse. When the volume of xenograft tumours reached 100–300 mm^3^, all rats were randomly divided into three groups: control group (*n* = 5), model group (*n* = 5), and treatment group (*n* = 5). We used vernier calipers to measure the longest and shortest diameter of the tumour every week, and the tumour volume (*V*), relative tumour volume (RTV) and tumour relative value-added rate (TRAR) were calculated thus: Volume (*V*) = (length × width^2^)/2; relative tumour volume (RTV) = *V*_T_/*V*_O_. *V*_T_: tumour volume measured at time *T, V*_O_ : tumour volume measured before the experiment; tumour relative value-added rate (TRAR) = T_RTV_/M_RTV_ × 100%, T_RTV_: RTV of treatment group, C_RTV_: RTV of mode group. The treatment group was treated with PAC suspension solution 1.6 g/kg intragastrically daily for 28 days. The control group and model group received the equivalent volume of 0.5% carboxymethyl cellulose solvent in the same way. Before the end of the experiment for 24 h all the experimental animals were fasted but had free to access water. All animal care and experimental procedures were performed in compliance with the Ethical Committee of Heilongjiang University of Chinese Medicine (approval number: HUCM2014-6037) and were conducted according to the principles expressed in the Declaration of Helsinki.

#### Biosample collection and preparation

##### PAC samples

The freeze-dried powder of PAC was accurately weighed to 0.28 g and suspended in 100 mL of 80% v/v methanol, and ultrasonically extracted for 30 min at room temperature. The extracted solution was centrifuged at 13,000 rpm for 15 minutes at 4 °C, both of the supernatants were filtered through a filter membrane (pore size: 0.22 μm), and then we injected 2 μL for constituent analysis based on UPLC-G2-Si-MS/MS.

#### Serum samples

On the 28th day blood collected from the eyeball and put into tubes and centrifuged at 3000 rpm at 5 °C for 15 min before collecting the serum for analysis. Some 800 μL of methanol was add to 50 μl serum samples, and extracted in an ultrasonic water bath for 1 min, from which we collected 800 μL supernatant and dried it under nitrogen gas at room temperature. Some 200 μL of methanol was used to redissolve the residues, then it was centrifuged at 13,000 rpm for 15 min at 4 °C. All samples were filtered through a filter membrane (pore size: 0.22 μm) before analysis, and then we injected 5 μL for constituent analysis and metabolomic analysis.

##### Tumour samples

On the 28th day, after all mice were sacrificed, we quickly harvested and weighed the tumours, storing them in 10% formalin for histopathology and immunohistochemical analyses. The inhibition rate of tumour growth was calculated using the following formula: tumour inhibition rate (TIR) = (M_W_ − T_W_)/M_W_ × 100%, M_W:_ the average weight of the mode group at the end of the experiment, T_W_: weight of treatment group at the end of the experiment.

### Histopathology, immunohistochemical, and TUNEL analyses

After 28 days, the nude mice were sacrificed and we harvested the tumour tissue immediately. We measured the tumour tissue mass, then quickly put the specimens into the 10% neutral-buffered formalin solution and fixed them for 12 h, and then the tissue samples were made into paraffin sections and stained with hematoxylin and eosin (H&E), and the tumour tissue morphology was observed under light microscopy. Motic Medical 6.0 software (Xiamen Motic Software Engineering Co., Ltd) was used for image acquisition (400x). Immunohistochemical (IHC) and TUNEL analyses were used to analyse the level of COX-2, PAS, AR, and Bcl-2 expressions and we observed the apoptosis of tumour cells in both model, and treatment, groups. The process of IHC analysis was as follows: a primary antibody was used to incubate the paraffin sections at 4 °C for 12 h before rinsing three times with PBS, we added the secondary antibody (COX-2, PAS, AR, and Bcl-2 monoclonal antibody) at 37 °C for 15 min and rinsed the samples three times with PBS, then we added the DAB chromogen at room temperature for 3 min before rinsing with distilled water, and then hematoxylin use was used as a counter-stain. We used Image-Pro Plus (IPP) software to quantify the integrated optical density (IOD) of COX-2, PAS, AR and Bcl-2. TUNEL analysis was performed according to the instructions supplied with the *In Situ* Cell Death Detection kit, POD (Roche; cat. No. 11684817910). From each group we selected five high-power fields to calculate the percentage rate of apoptotic cells for every 200 cells with the following formula: apoptosis rate = number of positive apoptotic cell/ 200 × 100%.

#### Metabolomic study

##### UPLC analysis

Chromatographic analysis was performed by Waters Acquity^TM^ ultra-performance LC system (Waters, Milford, MA, USA). Masslynx (V4.1) was used as the control software. 5 μL serum samples were separated by ACQUITY UPLC BEH C18 column (2.1 × 100 mm, 1.7 um, Waters Corporation, Milford, USA) at 35 °C and a flow rate of 0.3 mL/min. The first-rank mobile phase consisted of A (CH_3_CN: HCOOH = 100:0.1) and B (H_2_O: HCOOH = 100:0.1). The linear elution gradient program was used as follows: 0–1.5 min, 2–16% A; 1.5–2 min, 16–20% A; 2–4 min, 20–60% A; 4–5 min, 60–70% A; 5–8 min, 70–70% A; 8–10 min, 70–99% A.

##### MS analysis

The UPLC system was coupled to a Waters Synapt ^TM^ G2-Si High Definition TOF Mass (HDMS) system (Waters, Milford, USA) equipped with an electrospray ionisation (ESI) source were used for sample analysis. The ESI source was performed in both positive and negative ion modes. The optimal MS conditions were set as follows: in positive mode, the capillary voltage was set to 2.6 kV with the sampling cone voltage at 40.0 V; in negative mode, the capillary voltage was set to 2.4 kV with the sampling cone voltage at 40.0 V. The MS source temperature was set to 110 °C, the desolvation gas temperature was set to 350 °C under a desolvation gas flow of 600 L/h. The parameters for the full-scan MS data were set to collect masses ranging from 100 to 2300 Da to acquire all mass peaks. Scans were of 0.3 s duration each. The collision energy of the low-energy scans and high-energy scans were set to 10–30 eV and 20–40 eV. Leucine-enkephalin was used to ensure accurate mass data during MS acquisition in centroid mode as controlled by Masslynx^TM^ (V4.1) software.

#### Multivariate statistical analysis

To improve the accuracy of compound identification and quantification, Progenesis QI 1.0 software (Nonlinear Dynamics, 2014, Version: 1.0) was used for data pre-processing: it could obtain a three-dimensional matrix processed by EZinfo 2.0 software for multivariate data analyses, including principal component analysis (PCA), partial least-squared discriminant analysis (PLS-DA), and orthogonal partial least-squared discriminant analysis (OPLS-DA). Pareto scaling transformation and data normalisation were applied to the data processing before multivariate statistical analysis. The VIP-plot was obtained from OPLS-DA, it can directly reflect the contribution rate of the change in metabolic profiling. The *P*-value was proportional to the contribution rate between the model group and control group, according to the Student *t*-test to select *P*-values of less than 0.05 and the variables far from the origin as metabolite biomarkers.

### Identification of biomarkers and metabolic pathways

The accurate molecular mass of metabolite biomarkers were obtained from S-plot list (VIP > 3), and then we confirmed the molecular structure using MassLynx software (Waters Corporation, Milford, USA). Based on the preliminary information about molecular mass and molecular structure, we checked the accurate mass and structure by retrieving the on-line databases of HMDB (http://www.hmdb.org/), ChemSpider (http://www.chemspider.com/), KEGG (http://www.genome.jp/ kegg/), METLIN (http://metlin.scripps.edu/), or Lipid Maps(http://dev.lipidmaps.org) with mass tolerances less than 5mDa. Finally the metabolite biomarkers were identified by comparing MS/MS fragment ions through MassFragment™ application manager analysis. The metabolite biomarkers were input to the MetaboAnalyst platform (http://www.metaboanalyst.ca/) to analyse the relative metabolic pathways which were associated with prostate cancer.

#### Constituent analysis *in vitro* and *in vivo*

##### UPLC analysis

Chromatographic analysis was performed on a Waters Acquity^TM^ ultra-performance LC system (Waters, Milford, MA, USA). Masslynx (V4.1) was used as the control software. 5 μL serum samples were separated by ACQUITY UPLC BEH C18 column (2.1 × 100 mm, 1.7 um, Waters Corporation, Milford, USA) at 35 °C and a flow rate of 0.3 mL/min. The first-rank mobile phase consisted of A (CH_3_CN: HCOOH = 100:0.1) and B (H_2_O: HCOOH = 100:0.1). The linear elution gradient program was used as follows: 0–1.5 min, 2–16% A; 1.5–2 min, 16–20% A; 2–4 min, 20–60% A; 4–5 min, 60–70% A; 5–8 min, 70–70% A; 8–10 min, 70–99% A.

##### MS analysis

The UPLC system was coupled with a Waters Synapt ^TM^ G2-Si High Definition TOF Mass (HDMS) system (Waters, Milford, USA) equipped with electrospray ionisation (ESI) source for sample analysis. The ESI source was performed in both positive and negative ion modes. The optimal MS conditions were set as follows: in positive mode, the capillary voltage was 2.6 kV with the sampling cone voltage at 40.0 V; in negative mode, the capillary voltage was 2.4 kV with the sampling cone voltage at 40.0 V. The MS source temperature was 110 °C, and the desolvation gas temperature was set at 350 °C at a flow rate of 600 L/h. The parameters for full-scan MS data were set to collect masses ranging from 100 to 2300 Da to acquire all mass peaks. Scans were of 0.3 s duration each. The collision energy of the low energy scans and high energy scans were set at 10–30 eV and 20–40 eV. Leucine-enkephalin was used for ensuring to obtain accurate mass data during MS acquisition in centroid mode as controlled by Masslynx^TM^ (V4.1) software.

#### Correlation analysis of marker metabolites and absorbed constituents

Under the premise that PAC could effectively inhibit the growth of xenograft tumours, we established a correlation between marker metabolites and serum constituents (PCMS), and then discovered the effective substances in PAC acting against prostate cancer. Firstly, we researched the standardisation of serum constituent multi-component chemical information and metabolite relative intensity. Then a correlation coefficient (*r*) between serum constituents and metabolites was calculated by Pearson’s correlation analysis method, this was used to determine the strength of the relationship between metabolite biomarkers and serum constituents, and revealed which serum constituents were the most related to therapeutic effect. In this study, *r* ranged from −1 to +1; *r* values greater than 0 meant that metabolite biomarkers and serum constituents were positively correlated, in contrast *r* values less than 0 meant that two variables were negatively correlated, and 0.9 < |r| ≤ 1 implied extremely well related, while 0.85 < |r| ≤ 1 implied highly related. The larger absolute value of *r*, the higher the correlation of metabolite biomarkers and serum constituents, and the greater their contribution to disease treatment.

#### Therapeutic target prediction of highly correlated ingredients

A professional High-Throughput Docking database (http://www.cbligand.org/HTDocking/) and PharmMapper database (http://lilab.ecust.edu.cn/pharmmapper/) were used to discover the relationships between the therapeutic targets and the chemical compounds. These on-line databases are designed by docking drug protein targets and then revealing the potential pharmacological effect of a small molecule and may give us biological information about target components, and helping the search for bioactive compounds which could effectively treat disease. In this study, on the premise that PAC could effectively inhibit the growth of xenograft tumours, we used a chinmedomics method to analyse the correlation between chemical components and metabolite biomarkers *in vivo*, and then selected the effective chemical components absorbed into blood based on |r| ≥ 0.85. Both of the candidate compounds were imported into the High-Throughput Docking, and PharmMapper databases, then we selected the protein targets with higher fitness scores as the top biological targets, and input these top protein targets into the KEGG database (http://www.genome.jp/ kegg/) to annotate and analyse the relevant pathway.

### Statistical analysis

The SPSS V19.0 data software package was used to statistically analyse the data pertaining to volume, mass, relative tumour volume, tumour relative value-added rate, and tumour inhibition rate. Results are shown as mean ± standard deviation (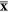
** ± s)**. Statistically significant differences in volume, mass, relative tumour volume, tumour relative value-added rate and tumour inhibition rate of the mean value between two groups were determined by Student’s *t*-test. The metabolite biomarkers were assessed by multivariate statistical analysis (VIP > 3 and *P* < 0.05). In addition, one-way analysis of variance (ANOVA) was used to analyse the expression levels of identified biomarkers in different groups. Statistical significance was taken as *P* < 0.05, and *P* < 0.01 was considered extremely significant.

## Additional Information

**How to cite this article:** Li, X.-N. *et al*. Screening the active compounds of *Phellodendri Amurensis* cortex for treating prostate cancer by high-throughput chinmedomics. *Sci. Rep.* **7**, 46234; doi: 10.1038/srep46234 (2017).

**Publisher's note:** Springer Nature remains neutral with regard to jurisdictional claims in published maps and institutional affiliations.

## Supplementary Material

Supplementary Information
