# Supplementary material for: Screening the active compounds of Phellodendri Amurensis cortex for treating prostate cancer by high-throughput chinmedomics
Source: Sci Rep. 2017 Apr 6;7:46234. doi: 10.1038/srep46234 (PMC5382783; doi:10.1038/srep46234)
Supplement: Supplementary Information [file srep46234-s1.doc]

**Screening the active compounds of *Phellodendri Amurensis cortex* for treating prostate cancer by high-throughput chinmedomics**

Xian-Na Li^‡^, Aihua Zhang^‡^, Meijia Wang, Hui Sun, Zhidong Liu, Shi Qiu, Tianlei Zhang, Xijun Wang.

Sino-America Chinmedomics Technology Collaboration Center, National TCM Key Laboratory of Serum Pharmacochemistry, Chinmedomics Research Center of TCM State Administration, Laboratory of Metabolomics, Department of Pharmaceutical Analysis, Heilongjiang University of Chinese Medicine, Heping Road 24, Harbin 150040, China.

*Address correspondence to:

Prof. Xijun Wang

Sino-America Chinmedomics Technology Cooperation Center, National TCM Key Laboratory of Serum Pharmacochemistry, Research Center of Chinmedomics (State Administration of TCM), Laboratory of Metabolomics, Heilongjiang University of Chinese Medicine, Heping Road, Harbin, China.

Tel. & Fax +86-451-82110818

Email: xijunwangtcm@yeah.net

^‡^ These authors contributed equally to this work.

**Table 1.The result of relative tumor volume (RTV), tumor relative value-added rate (TRAR) and tumor inhibition rate (TIR) in model group and treatment group (±S, n=5)**

| **Time(d)** | **Model group** | **Treatment group** | | |
| --- | --- | --- | --- | --- |
|  | **RTV**(mm^3^) | **RTV**(mm^3^) | **TRAR**(%) | **TIR**(%) |
| 7 | 4.1±1.1 | 1.6±0.3 | 38.8^**^ | —— |
| 14 | 8.5±4.3 | 2.4±0.5 | 28.4^*^ | —— |
| 21 | 14.3±3.9 | 4.9±3.3 | 34.5^**^ | —— |
| 28 | 25.3±8.2 | 8.2±5.5 | 32.3^**^ | 62.8^**^ |

*：P＜0.05 ；**：P＜0.01 vs model group

**Table 2.**The IOD value of PSA, AR, COX-2, Bcl-2 and Caspase-3 in model group and treatment group (±S, n=5)

| **Group** | **PSA** | **AR** | **COX-2** | **Bcl-2** | **Caspase-3** |
| --- | --- | --- | --- | --- | --- |
| Model group | 0.34±0.09 | 0.33±0.08 | 0.29±0.02 | 0.28±0.01 | 0.18±0.06 |
| Treatment group | 0.21±0.02* | 0.24±0.02* | 0.25±0.02* | 0.25±0.02* | 0.26±0.02* |

*：P＜0.05 vs model group

**Table 3.** Candidate biomarkers in prostate cancer model nude mouse identified by UPLC-G2-Si-MS/MS.

| **No.** | **Retention Time** | | **Mass** | **Calc.**  **Mass** | **[H-H]-/**  **[H+H]^+^** | **ppm** | **Formular** | **Mass Fragments** | **Identified** | **Trend** | **Metabolomic**  **pathway** |
| --- | --- | --- | --- | --- | --- | --- | --- | --- | --- | --- | --- |
| 1 | 0.73 | 191.0770 | | 191.0780 | [M+H]^+^ | -3.1 | C_6_H_14_N_4_O_3_ | 175[M+H-H_2_N]^+^;131[M+H-CH_4_N_2_O]^+^; | N-(ω)-Hydroxyarginine | ↓ | Arginine and proline metabolism |
| 2 | 0.86 | 169.0365 | | 169.0362 | [M+H]^+^ | 2.4 | C_5_H_4_N_4_O_3_ | 169[M+H]^+^;152[M+H-H_3_N]^+^;141[M+H-CO]^+^; | Uric acid | ↑ | Purine metabolism |
| 3 | 1.25 | 111.0080 | | 111.0082 | [M-H]^-^ | -3.6 | C_5_H_4_O_3_ | 111[M-H]^-^ | 2-Furoic acid | ↑ | Furfural degradation |
| 4 | 1.25 | 191.0184 | | 191.0192 | [M-H]^-^ | -4.7 | C_6_H_8_O_7_ | 112[M-H]^-^;87[M-H-C_3_H_4_O_4_]^-^;85[M-H-C2H_2_O_5_]^-^ | Isocitric acid | ↑ | Citrate cycle |
| 5 | 1.50 | 180.0653 | | 180.0661 | [M-H]^-^ | -5.0 | C_9_H_11_NO_3_ | 180[M-H]^-^;163[M-H-H_3_N]^-^;119[M-H-CH_3_NO_2_]^-^ | Beta-Tyrosine | ↓ | Tyrosine metabolism |
| 6 | 1.50 | 165.0550 | | 165.0552 | [M+H]^+^ | 1.8 | C_9_H_8_O_3_ | 165[M+H]^+^;147[M+H-H_2_O]^+^;119[M+H-CH_2_O_2_]^+^; | 2-Hydroxycinnamic acid | ↓ | Biosynthesis of secondary metabolites |
| 7 | 4.18 | 415.1975 | | 415.1977 | [M-H]^-^ | -0.2 | C_19_H_30_O_5_S | 415[M+HCOO]^-^;351[M+HCOO-H_2_O]^-^; | Androsterone sulfate | ↑ | Steroid hormone biosynthesis |
| 8 | 4.45 | 329.2324 | | 329.2328 | [M-H]^-^ | -1.8 | C_18_H_34_O_5_ | 329[M-H]^-^;229[M-H-C_6_H_12_O]^-^;211[M-H-C_6_H_14_O_2_]^-^; | 9,12,13-TriHOME | ↓ | Linoleic acid metabolism |
| 9 | 4.84 | 333.2061 | | 333.2066 | [M-H]^-^ | -0.3 | C_20_H_30_O_4_ | 333[M-H]^-^;316[M-H-HO]^-^;285[M-H-CH_4_O_2_]^-^; | Prostaglandin A2 | ↓ | Arachidonic acid metabolism |
| 10 | 4.89 | 335.2226 | | 335.2222 | [M-H]^-^ | 0.9 | C_20_H_32_O_4_ | 335[M-H]^-^;275[M-H-C_2_H_4_O_2_]^-^;179[M-H-C_9_H_16_O_2_]^-^ | Prostaglandin A1 | ↓ | Arachidonic acid metabolism |
| 11 | 5.59 | 301.2168 | | 333.2168 | [M+H]^+^ | 0.0 | C_20_H_28_O_2_ | 301[M+H]^+^;201[M+H-C_6_H_12_O]^+^;145[M+H-C_9_H_16_O_2_]^+^; | All-trans-retinoic acid | ↓ | Retinol metabolism |
| 12 | 5.64 | 378.2415 | | 378.2409 | [M-H]^-^ | 2.6 | C_18_H_38_NO_5_P | 335[M-H-C_3_H_7_]^-^;96[M-H-C_18_H_35_NO]^-^; | Sphingosine 1-phosphate | ↓ | Sphingolipid metabolism |
| 13 | 5.84 | 518.3249 | | 518.3247 | [M+H]^+^ | 0.4 | C_26_H_48_NO_7_P | 518[M+H]^+^;184[M+H-C_17_H_37_NO_3_P]^+^; | LysoPC(18:3(6Z,9Z,12Z)) | ↑ | Glycerophospholipid metabolism |
| 14 | 6.04 | 295.2278 | | 295.2273 | [M-H]^-^ | 1.7 | C_18_H_32_O_3_ | 277[M-H-H_2_O]^-^;251[M-H-C_2_H_4_O]^-^ | 13S-hydroxyoctadecadienoic acid | ↑ | Linoleic acid metabolism |
| 15 | 6.17 | 880.5179 | | 880.5155 | [M-H]^-^ | 1.8 | C_52_H_84_NO_8_P | 880[M-H]^-^；862[M-H-H_2_O]^-^ | PC(22:6(4Z,7Z,10Z,13Z,16Z,19Z)/22:4(7Z,10Z,13Z,16Z)) | ↓ | Choline metabolism in cancer |
| 16 | 6.18 | 313.1804 | | 313.1804 | [M-H]^-^ | 0.0 | C_20_H_26_O_3_ | 313[M-H]^-^;269[M-H-CO_2_]^-^ | 4-oxo-Retinoic acid | ↓ | Retinol metabolism |
| 17 | 6.24 | 564.5305 | | 564.5315 | [M-H]^-^ | -1.8 | C_36_H_71_NO_3_ | 564[M-H]^-^;281[M-H-C_19_H_39_O]^-^;242[M-H-C_23_H_46_]^-^; | Ceramide (d18:1/18:0) | ↑ | Sphingolipid metabolism |
| 18 | 6.39 | 398.3329 | | 398.3316 | [M+H]^+^ | 3.3 | C_22_H_39_NO_5_ | 398[M+H]^+^;319[M+H-C_3_H_13_NO]^+^;312[M+H-C_5_H_10_O]^+^; | PGF2a ethanolamide | ↑ | Sphingolipid metabolism |
| 19 | 6.74 | 184.0744 | | 184.0738 | [M+Na]^+^ | 3.3 | C_10_H_11_NO | 184[M+Na]^+^;98[M+Na -C_2_H_9_NO]^+^; | Tryptophanol | ↑ | Tryptophan metabolism |
| 20 | 6.81 | 319.2284 | | 319.2273 | [M-H]^-^ | 3.4 | C_20_H_32_O_3_ | 319[M-H]^-^;301[M-H-H_2_O]^-^;257[M-H-CH_2_O_3_]^-^; | 16(R)-HETE | ↑ | Arachidonic acid metabolism |
| 21 | 6.81 | 303.2335 | | 303.2324 | [M+H]^+^ | 3.6 | C_20_H_30_O_2_ | 303[M+H]^+^;145[M+H-C_9_H_18_O_2_]^+^; | Eicosapentaenoic acid | ↓ | Biosynthesis of unsaturated fatty acids |
| 22 | 7.07 | 482.362 | | 482.3612 | [M+H]^+^ | 2.1 | C_30_H_59_NO_3_ | 482[M+H]^+^;184[M+H-C_18_H_36_NO_2_]^+^; | Ceramide (d18:1/12:0) | ↓ | Sphingolipid metabolism |
| 23 | 7.31 | 250.1438 | | 250.1416 | [M-H]^-^ | -2.0 | C_10_H_13_N_5_O_3_ | 235[M-H-O]^-^;205[M-H-NO_2_]^-^ | 5'-Deoxyadenosine | ↓ | Unknown |
| 24 | 7.37 | 508.3773 | | 508.3764 | [M+H]^+^ | 1.8 | C_26_H_54_NO_6_P | 508[M+H]^+^;166[M+H-C_21_H_42_O_3_]^+^;98[M+H-C_23_H_55_NO_4_]^+^; | LysoPC(P-18:0) | ↓ | Glycerophospholipid metabolism |
| 25 | 7.61_ | 599.3197 | | 599.3198 | [M-H]^-^ | 0.2 | C_40_H_56_O_4_ | 283[M-H-C_19_H_24_O_4_]^-^;241[M-H-C_21_H_42_O_4_]^-^; | Neoxanthin | ↑ | Biosynthesis of secondary metabolites |
| 26 | 7.98 | 568.3622 | | 568.3625 | [M-H]^-^ | -0.5 | C_30_H_52_NO_7_P | 568[M-H]^-^;509[M-H-C_2_H_3_O_2_]^-^;285[M-H-C_9_H_18_NO_7_P]^-^; | LysoPC(22:5(4Z,7Z,10Z,13Z,16Z)) | ↑ | Glycerophospholipid metabolism |
| 27 | 8.29 | 482.3259 | | 482.3247 | [M+H]^+^ | 2.5 | C_23_H_48_NO_7_P | 482[M+H]^+^;464[M+H-H_2_O]^+^;342[M+H-C_2_H_7_NO_4_P]^+^; | LysoPC(15:0) | ↓ | Glycerophospholipid metabolism |
| 28 | 8.36 | 506.3613 | | 506.3610 | [M+H]^+^ | 0.6 | C_26_H_52_NO_6_P | 506[M+H]^+^;447[M+H-C_3_H_9_N]^+^;163[M+H-C_21_H_45_NO_2_]^+^; | LysoPC(P-18:1(9Z)) | ↑ | Glycerophospholipid metabolism |
| 29 | 10.04 | 303.2327 | | 303.2324 | [M-H]^-^ | 1.0 | C_20_H_32_O_2_ | 285[M-H-H_2_O]^-^;260[M-H-CO_2_]^-^;231[M-H-C_3_H_4_O_2_]^-^; | Arachidonic acid | ↑ | Arachidonic acid metabolism |
| 30 | 10.34 | 376.3180 | | 376.3216 | [M+H]^+^ | -9.6 | C_24_H_41_NO_2_ | 376[M+H]^+^;151[M+H-C_15_H_29_O]^+^; 124[M+H-C_17_H_31_O]^+^; | Adrenoyl ethanolamide | ↓ | Sphingolipid metabolism |
| 31 | 10.35 | 279.2324 | | 279.2324 | [M-H]^-^ | 0.0 | C_18_H_32_O_2_ | 261[M-H-H_2_O]^-^;251[M-H-C_2_H_4_]^-^;114[M-H-C_12_H_21_]^-^ | Linoleic acid | ↑ | Linoleic acid metabolism |
| 32 | 10.80 | 813.6748 | | 813.6751 | [M+H]^+^ | -0.4 | C_47_H_93_N_2_O_6_P | 813[M+H]^+^;184[M+H-C_38_H_80_NO_3_P]^+^; | SM(d18:1/24:1(15Z)) | ↑ | Sphingolipid metabolism |
| 33 | 10.98 | 303.3037 | | 303.3028 | [M+Na]^+^ | 3.0 | C_20_H_40_Na | 303[M+Na]^+^;219[M+Na-C_4_H_14_]^+^ | 8-Isoprostane | ↓ | Arachidonic acid metabolism |
| 34 | 11.40 | 319.2986 | | 319.2977 | [M+Na]^+^ | 2.8 | C_20_H_40_O | 319[M+Na]^+^;109[M+Na-C_12_H_28_O]^+^;95[M+Na-C_13_H_30_O]^+^ | Thromboxane | ↓ | Arachidonic acid metabolism |

**Table 4.**Results of metabolic pathways analysis based on KEGG database

| No. | | Pathway Name | Total | Hits | P | -log(p) | Holm p | FDR | [Impact](http://www.metaboanalyst.ca/MetaboAnalyst/faces/Secure/Pathway/ResultView.jsp?form1:table1:tableRowGroup1:tableColumn6:_columnHeader:_toggleSortButton_submittedLink=form1:table1:tableRowGroup1:tableColumn6:_columnHeader:_toggleSortButton) |
| --- | --- | --- | --- | --- | --- | --- | --- | --- | --- |
| 1 | Linoleic acid metabolism | | 6 | 0.12279 | 3 | 0.000148 | 8.8173 | 0.012148 | 0.012148 |
| 2 | Arachidonic acid metabolism | | 36 | 0.73677 | 4 | 0.005302 | 5.2397 | 0.42944 | 0.21737 |
| 3 | Sphingolipid metabolism | | 21 | 0.42978 | 3 | 0.008006 | 4.8276 | 0.64045 | 0.21882 |
| 4 | Biosynthesis of unsaturated fatty acids | | 42 | 0.85956 | 3 | 0.051809 | 2.9602 | 1 | 1 |
| 5 | Glycerophospholipid metabolism | | 30 | 0.61397 | 2 | 0.1237 | 2.0899 | 1 | 1 |
| 6 | alpha-Linolenic acid metabolism | | 9 | 0.18419 | 1 | 0.17025 | 1.7705 | 1 | 1 |
| 7 | Retinol metabolism | | 16 | 0.32745 | 1 | 0.28297 | 1.2624 | 1 | 1 |
| 8 | Glyoxylate and dicarboxylate metabolism | | 18 | 0.36838 | 1 | 0.31235 | 1.1636 | 1 | 1 |
| 9 | Citrate cycle (TCA cycle) | | 20 | 0.40932 | 1 | 0.34058 | 1.0771 | 1 | 1 |
| 10 | Arginine and proline metabolism | | 44 | 0.90049 | 1 | 0.6031 | 0.50567 | 1 | 1 |
| 11 | Purine metabolism | | 68 | 1.3917 | 1 | 0.76327 | 0.27015 | 1 | 1 |
| 12 | Steroid hormone biosynthesis | | 72 | 1.4735 | 1 | 0.783 | 0.24463 | 1 | 1 |

Note: Total: the total number of the compound in metabolism pathways; Hits: the number of exact matches in the uploaded data; Raw P: the original P value through the pathway analysis. Impact: the pathway impact value through topological analysis.

**Table 5. MS and MS/MS data of the identified components of *Phellodendri Amurensis* Cortex.**

| **No.** | **t_R_** | **Formula** | **Obsd**  **[M+H]+/ [M-H]^-^** | **Calcd**  **[M+H]+/ [M-H]^-^** | **Error**  **/ppm** | **Fragment ions** | **Identify** |
| --- | --- | --- | --- | --- | --- | --- | --- |
| 1* | 0.51 | C_19_H_18_O_6_ | 341.1110 | 341.111 | -0.9 | 341[M-H]^-^;179[M-H-C_10_H_10_O]^-^;161[M-H-C_9_H_8_O_4_]^-^;89[M-H-C_13_H_16_O_5_]^-^ | Tetramethyl-O-scutellarin |
| 2 | 1.71 | C_11_H_18_NO | 180.1397 | 180.1388 | 5.0 | 180[M]^+^;121[M-C_3_H_9_N]^+^;120[M-C_3_H_10_N]^+^; | N-candicine |
| 3* | 1.82 | C_13_H_16_O_10_ | 331.0675 | 331.0665 | 3.0315 | 331[M-H]^-^;313[M-H-H_2_O]^-^;168[M-H- C_6_H_10_O_4_]^-^;149[M-H-H_2_O-H]^-^；125[M-C_7_H_4_O_5_-2H_2_O]^-^; | b- C_6_H_10_O_5_cogallin |
| 4* | 2.16 | C_23_H_30_O_14_ | 529.1565 | 529.1557 | 1.5 | 529[M-H]^-^;484;355[M-H-C_7_H_10_O_5_]^-^;193[M-H-C_7_H_10_O_5_- C_6_H_10_O_4_]^-^;178[M-H-C_7_H_10_O_5_- C_6_H_10_O_4_ -CH_3_]^-^;173;149[M-H- C_6_H_10_O_5_- C_8_H_10_O_7_]^-^;134[M-H- C_6_H_10_O_5_-C_8_H_10_O_7_-CH_3_]^-^;111 | 3-({3-[4-(β- C_6_H_10_O_5_copyranosyloxy)-3-methoxyphenyl]-2-propenoyl}oxy)-1;4;5-trihydroxycyclohexanecarboxylic acid |
| 5* | 2.01 | C_13_H_16_O_9_ | 315.0732 | 315.0716 | 1.6 | 315[M-H]^-^;153[M-H- C_6_H_10_O_5_]^-^ | 3-Carboxy-4-hydroxy-phenoxy C_6_H_10_O_5_coside |
| 6* | 2.14 | C_26_H_30_O_12_ | 533.1516 | 533.1515 | 0.2 | 533[M-H]^-^;324[M-H-C_12_H_16_O_3_]^-^;192[M-H-C_14_H_28_O_9_]^-^；178[M-H-C_17_H_22_O_8_]^-^; | Amurensin |
| 7* | 2.28 | C_23_H_30_O_14_ | 529.1578 | 529.1557 | 4.0 | 529[M-H]^-^;367[M-H- C_6_H_10_O_5_]^-^;191[M-H- C_6_H_10_O_5_-C_10_H_8_O_3_]^-^;173[M-H- C_6_H_10_O_5_-C_10_H_11_O_4_]^-^; | 3-O-feruloylquinicacid C_6_H_10_O_5_coside |
| 8* | 2.12 | C_9_H_10_O_5_ | 197.0466 | 197.0450 | 1.6 | 197[M-H]^-^;153[M-H-COO-]^-^ | 2;3;4-Trihydroxy-benzenepropanoic acid |
| 9* | 2.29 | C_16_H_18_O_9_ | 353 | 314;1756 | 1.3 | 353[M-H]^-^;191[M-C_9_H_6_O_3_]^-^ | Neochlorogenic acid |
| 10* | 2.4 | C_19_H_28_O_11_ | 431.1564 | 431.1553 | 2.6 | 431[M-H]^-^;299[M-H-C_5_H_8_O_4_]^-^;191[M-H- C_6_H_10_O_4_ -C_6_H_6_O]^-^；149[M-H- C_6_H_10_O_4_ -C_8_H_8_O]^-^; | 2-(p-hydroxyphenyl) ethanol 1-O-β-D- C_6_H_10_O_5_coside |
| 11 | 2.7 | C_19_H_24_NO_3_ | 314.1749 | 314.1756 | -2.2 | 314[M]^+^;238[M-C_4_H_14_N]^+^;237.0[M-C_4_H_15_N]^+^;225[M-C_5_H_15_N]^+^;209[M-C_7_H_5_O]^+^;175[M-C_9_H_15_O]^+^;107[M-C_12_H_17_NO_2_]^+^;58[M-C_16_H_16_O_3_]^+^; | (-)-Oblongine |
| 12* | 2.56 | C_16_H_18_O_9_ | 353.0882 | 353.0873 | 2.5 | 239[M-H-C_4_H_12_N]^-^;224[M-H-C_5_H_14_N]^-^;191[M-H-C_8_H_10_O]^-^;105[M-H-C_13_H_21_NO]^-^; | Chlorogenic acid |
| 13 | 2.79 | C_23_H_30_NO_8_ | 448.1997 | 448.1971 | 2.6 | 448[M+H]^+^;286[M+H-C_6_H_10_O_4_]^+^;255[M+H- C_6_H_10_O_4_ -CH_2_OH]^+^; | (p-hydroxybenzyl)-6; 7-dihydroxy-N-m ethyltetrahydro iso- quinoline-7-O-p--D- C_6_H_10_O_5_copyranoside |
| 14* | 2.81 | C_10_H_10_O_4_ | 193.0513 | 193.0501 | 1.2 | 284[M-H- C_6_H_10_O_5_]^-^; | Ferulic acid |
| 15 | 2.40 | C_17_H_19_O_9_ | 369.1160 | 369.1186 | -2.6 | 369[M+H]^+^;193[M+H-C_10_H_8_O_3_] ^+^; | Amurenlactone A/B |
| 16 | 2.85 | C_20_H_24_NO_4_ | 342.1733 | 342.1705 | 2.8 | 406[M+H-OH]^+^;371[M+H-CH_8_O_2_]^+^;303[M+H-C_4_H_8_O_4_]^+^;273[M+H-C_7_H_2_O_4_]^+^;245[M+H-C_7_H_14_O_5_]^+^; | Phellodendrine |
| 17 | 2.96 | C_36_H_34_NO_7_ | 592 .2332 | 592.2335 | -0.5 | 592[M+H]^+^; | unknown |
| 18 | 3.04 | C_20_H_26_NO_4_ | 344.1866 | 344.1862 | 1.2 | 273[M-C_4_H_10_N]^+^;255[M-C_5_H_16_N]^+^;192[M-C_9_H_13_0_2_]^+^;177[M-C_9_H_13_O_2_-CH_3_]^+^; | Tembetarine |
| 19 | 3.03 | C_20_H_24_NO_4_ | 342.1733 | 342.1705 | 2.8 | 327[M-CH_3_]^+^[M-3(CH_3_)]^+^;295[M-C_3_H_11_]^+^;282[M-C_3_H_10_N]^+^;266[M-C_4_H_14_N]^+^;237[M-C_5_H_15_NO]^+^; | Magnoflorine |
| 20 | 3.06 | C_19_H_22_NO_4_ | 328.2 | 314.1756 | -2.9 | 328[M]^+^ | Terahydroreticuline |
| 21 | 3.06 | C_16_H_20_NO_3_ | 274.1421 | 274.143 | -4.4 | 256[M-H_2_O]^+^;226[M-H_2_O-2CH_3_]^+^;202[M-OCH_3_-C_2_H_4_]^+^;172[M-OCH_3_-C_2_H_4_-OCH_3_]^+^; | unknown |
| 22* | 3.23 | C_16_H_20_O_10_ | 371.0983 | 371.0978 | 1.3 | 371[M-H]^-^;296;193;195[M-H-( C_6_H_10_O_5_-H_2_O)]^-^; | Syringin |
| 23 | 3.15 | C_14_H_14_N_7_O_2_ | 312.1205 | 312.1209 | -1.3 | 312[M+H]^+^;297[M+H-CH_3_]^+^;268[M+H-CH_3_-C_2_H_5_]^+^; | unknowm |
| 24 | 3.14 | C_20_H_22_NO_4_ | 340.1499 | 340.1549 | -5.0 | 340[M]^+^;325[M-CH_3_]^+^;308[M-CH_3_-OH]^+^;294[M-CH_3_-CH_2_O]^+^;262[ M-CH_3_-CH_2_O-CH_3_OH]^+^; | 9;10-Dimethpey-7;8;13;13a-tetradroberbinium |
| 25 | 3.17 | C_20_H_23_NO_4_ | 342.1653 | 342.1665 | -3.5 | 311[M+H-C_2_H_7_]^+^;310[M+H-C_2_H_8_]^+^;297[M+H-3CH_3_]^+^;284[M+H-2CH_3_-C_3_H_4_]^+^; | Tetrahydrojatrorrhizine |
| 26* | 3.12 | C_17_H_20_O_9_ | 367.1160 | 367.1186 | -2.6 | 367[M-H]^-^;353[M-H-CH_2_]^-^;193[M-H-C_10_H_6_O_3_]^-^;191[M-H-C_10_H_8_O_3_]^-^;189[M-H-C_10_H_10_O_3_]^-^; | 3-O-feruloylquinic acid |
| 27* | 3.20 | C_21_H_22_NO_4_ | 356.1839 | 356.1862 | -2.3 | 356[M]^+^;312[M-C_3_H_7_]^+^;311[M-C_2_H_6_N] ^+^;298[M-C_4_H_9_]^+^;296[M-C_4_H_11_]^+^;279[M-C_4_H_12_O]^+^; | Tetrahydropalmatine |
| 28 | 3.21 | C_40_H_38_NO_3_ | 581．2232 | 581.2234 | -0.3 | 581[M-H]^-^;419[M-H- C_6_H_10_O_5_]^-^;373[M-H- C_6_H_10_O_5_-C_2_H_5_OH]^-^; | (±)-5;5’-dimethoxylaricire-sinol-4-O- C_6_H_10_O_5_coside |
| 29* | 3.28 | C_19_H_24_NO_3_ | 314.1749 | 314.1756 | -2.2 | 315[M+H]^+^;271[M+H-C_3_H_8_]^+^;268[M+H-C_3_H_11_]^+^;209[M+H-C_7_H_6_O]^+^;107[M+H-C_12_H_18_NO_2_]^+^; | Lotusine |
| 30 | 3.28 | C_17_H_22_O_10_ | 385.1138 | 385.1135 | 0.8 | 209[M-H-( C_6_H_10_O_5_-H_2_O) ]^-^;193[M-H- C_6_H_10_O_5_-OH]^-^；134[M-H-CH_3_-C_8_H_10_O_7_-H_2_O]^-^; | 3-hydroxyl feruloylquinic acid |
| 31* | 3.35 | C_27_H_36_O_13_ | 567．2072 | 567.2078 | -1.1 | 567[M-H+HCOOH]^-^;521[M-H]^-^;341[M-H- C_6_H_10_O_4_ -H_2_O]^-^;329[M-H- C_6_H_10_O_4_ -OCH_3_]-;162[C_6_H_10_O_5_]^-^ | (+/-)8-(4-Hydroxy-3-methoxyphenyl)-6;7-bis(hydroxymethyl)-3-methoxy-5;6;7;8-tetrahydro-2-naphthalenyl β-D- C_6_H_10_O_5_copyranoside |
| 32 | 3.44 | C_21_H_25_NO_4_ | 356.1839 | 356.1862 | -2.3 | 356[M]^+^;325[M-C_2_H_7_]^+^;294[M-C_4_H_14_]^+^;293[M-C_4_H_15_]^+^;232[M-C_7_H_8_O_2_]^+^;192[M-C_10_H_12_O_2_]^+^; | Menisperine |
| 33 | 3.45 | C_21_H_24_NO_4_ | 463.0878 | 463.9877 | 0.2 | 340[M-CH_3_]^-^;322[M-2CH_3_]^-^;190[M-C_10_H_13_O_2_]^-^ | N-Methyl canadine |
| 34 | 3.48 | C_19_H_22_NO_4_ | 328.1913 | 328.1872 | 3.6 | 328[M+H]^+^;283[M+H-CH_3_CH_2_O]^+^;251[M+H-CH_3_CH_2_O-CH_3_OH]^+^;223[M+H-CH_3_CH_2_O-CH_3_OH-CO]^+^; | Armepavine |
| 35 | 3.52 | C_17_H_21_NO_4_ | 304.1529 | 304.1549 | -2.0 | 304[M+H]^+^;255[M+H-C_2_H_9_O]^+^;239[M+H-C_3_H_13_O]^+^;232[M+H-C_5_H_12_]^+^;206[M+H-C_6_H_10_O]^+^; | Dasycarpamin |
| 36 | 3.6 | C_21_H_22_NO_4_ | 356.1839 | 356.1862 | -2.3 | 356[M]^+^;312[M-C_3_H_7_]^+^;298[M-C_4_H_9_]^+^;296[M-C_4_H_11_]^+^;279[M-C_4_H_12_O]^+^;264[M-C_5_H_15_O]^+^; | Tetrahydropalmatine |
| 37 | 3.60 | C_20_H_18_NO_4_ | 352.1183 | 352.1185 | -0.6 | 352[M+H]^+^;336[M+H-CH_3_]^+^;322[M+H-2CH_3_]^+^;320[M+H-CH_4_O]^+^;308[M+H-CH_4_O-CH_3_]^+^; | Oxyberberine |
| 38 | 3.65 | C_26_H_32_O_8_ | 338.1405 | 338.1406 | -0.3 | 323[M-CH_3_]^+^;322[M –CH_4_]^+^;308[M-2CH_3_]^+^;307[M -2CH_3_-H]^+^;291[M-C_3_H_10_]^+^;290[M-C_3_H_12_]^+^; | Columbamine |
| 39 | 3.88 | C_18_H_34_O_5_ | 370.1985 | 370.1991 | -1.6 | 338[M-2CH_3_]^+^; 237[M-2CH_3_-C_5_H_9_O_2_]^+^; 206[M-CH_3_-C_9_H_7_O2]^+^; | 1;2;9;10-Tetramethoxy-6;6-dimethyl-5;6;6a;7-tetrahydro-4H-dibenzoquinolinium |
| 40 | 3.84 | C_26_H_30_O_7_ | 352.1592 | 352.1594 | -0.6 | 352[M]^+^;336[M-CH_4_]^+^;322[M-CH_3_-CH_3_]^+^;307[M-3CH_3_]^+^;294[M-CH_3_-CO-CH_4_]^+^; | Palmatine |
| 41 | 4.02 | C_21_H_22_NO_4_ | 354.1687 | 354.1705 | -5.1 | 354[M]^+^;294[M-C_2_H_4_O_2_]^+^;191[M-C_10_H_11_O_2_]^+^;190[M-C_10_H_12_O_2_]^+^;179[M-C_11_H_11_0_2_]^+^; | N-Methyl canadine |
| 42 | 4.05 | C_20_H_18_NO_4_ | 336.1211 | 336.1236 | -2.5 | 336[M]^+^;320[M-CH_4_]^+^;309[M-C_2_H_3_]^+^;306[M-CH_3_-CH_3_]^+^;292[M-CHO_2_]^+^;278[M-C_2_H_3_O_2_]^+^; | Berberine |
| 43 | 4.07 | C_26_H_30_O_9_ | 338.1405 | 338.1406 | -0.3 | 370[M]^+^;338[M-C_2_H_8_]^+^;321[M-C_3_H_13_]^+^;307[M-C_4_H_15_]^+^;279[M-C_5_H_17_N]^+^;222[M-C_9_H_8_O_2_]^+^; | Jatrorrhizine |
| 44 | 4.28 | C_26_H_30_O_8_ | 340.1580 | 340.1584 | -1.2 | 340[M+H]^+^;325[M+H-CH_3_]^+^;324[M+H-CH_4_]^+^;322[M+H-CH_6_]^+^;307[M+H-C_2_H_9_]^+^;296[M+H-CO_2_]^+^; | Tetrahydroberberine |
| 45* | 4.31 | C_19_H_34_O_15_ | 501.1743 | 501.1715 | 5.6 | 457[M-CO_2_]^-^;413[M-2CO_2_]^-^;395[M-2CO_2_-H_2_0]^-^；371[M-2CO_2_-H_2_0-CH_3_]^-^; | γ-hydroxybutenolide deniatives Ⅱ |
| 46* | 4.58 | C_18_H_34_O_5_ | 329.2352 | 328.2328 | 2.4 | 311[M-H-HO]^-^;293[M-H-2HO]^-^;229[M-H-HO-C_6_H_10_O]^-^;211[M-H-CO_2_-(CH_2_)_4_CH_3_]^-^; | Sanleng acid |
| 47* | 4.63 | C_26_H_30_O_9_ | 485.1815 | 485.1812 | 0.6 | 485[M-H]^-^;467[M-H-H_2_O]^-^;423[M-H-H_2_O-CO_2_]^-^;411[M-H-H_2_O-C_2_O_2_]^-^; | Rutaevin |
| 48* | 4.83 | C_26_H_30_O_8_ | 515.1984 | 515.1984 | 0.0 | 515[M-H+HCOOH]^-^;469[M-H]^-^; | Obaculactone |
| 49 | 4.83 | C_26_H_30_O_8_ | 471.1991 | 471.2019 | -5.9 | 471[M+H]^+^;453[M+H-H_2_0]^+^;425[M+H-CO-H_2_O]^+^;339[M+H-C_5_H_8_O_4_]^+^;161[M+H-C_16_H_22_O_6_]^+^; | Obaculactone |
| 50* | 5.04 | C_26_H_32_O_8_ | 471.1989 | 471.1960 | 3.1 | 471[M-H]^-^;413[M-H-C_3_H_6_O]^-^;383[M-H-C_4_H_8_O_2_]^-^;362[M-H-C_5_HO_3_]^-^;326[M-H-C_6_H_9_O_4_]^-^; | Obacunoic acid |
| 51 | 4.85 | C_21_H_22_NO_4_ | 352.1189 | 352.1194 | 2.6 | 351[M-H]^+^;336[M-CH_4_]^+^;322[M-CH_3_-CH_3_]^+^;294[M-C_3_H_9_N]^+^ | Thalphenine |
| 52 | 6.81 | C_26_H_30_O_7_ | 455.2030 | 455.2070 | -4.0 | 377[M+H-C_4_H_14_O]^+^;331[M+H-C_6_H_4_O_3_]^+^;315[M+H-C_7_H_8_O_3_]^+^;161[M+H-C_17_H_26_O_4_]^+^;94[M+H-C_21_H_28_O_5_]^+^ | Obacunone |
| 53* | 5.89 | C_31_H_27_O | 415.2064 | 415.2062 | 0.5 | 499[M+HCOOH]^-^;435[M-H_2_O]^-^;391[M-CO_2_-H_2_O]^-^;325[M-H-CH_3_]^-^;310[M-H-CH_3_-CH_3_]^-^; | T-Dehydrosigmasteroβ-sitostorol |
| 54* | 6.76 | C_7_H_27_N_12_O | 295.2296 | 295.2292 | 1.4 | 295[M-H]^-^;277[M-H-H_2_O]^-^; | unknown |

Note: Gal: galactose; Glu：Glucose;Glc: β-D-glucose; Rha: α-L-rhamnose;

**Table 6. MS and MS/MS data of the components identified in nude mouse plasma after oral administration of Phellodendri Amurensis Cortex.**

| **t_R_** | **Formula** | **Obsd**  **[M+H]+/ [M-H]^-^** | **Calcd**  **[M+H]+/ [M-H]^-^** | **Error**  **/ppm** | **Fragment ions** | **Identify** |
| --- | --- | --- | --- | --- | --- | --- |
| 1.71 | C_11_H_18_NO | 180.1397 | 180.1388 | 5.0 | 180[M]^+^;121[M-C_3_H_9_N]^+^;120[M-C_3_H_10_N]^+^; | N-candicine |
| 1.91 | C_24_H_28_NO_9_ | 474.0675 | 474.0665 | 3.0 | 298[M-(GluA-H_2_O)]^+^;283[ M-(GluA-H_2_O)-CH_3_]^+^;190[M-(GluA-2H_2_O)-2CH_3_-CH_3_COOH-]^+^; | M1(Hydroxymethyl Armepavine-O-Glucuronide) |
| 2.13 | C_24_H_28_NO_9_ | 474.0712 | 474.0665 | 1.7 | 298[M-(GluA-H_2_O)]^+^;283[ M-(GluA-H_2_O)-CH_3_]^+^;190[M-(GluA-2H_2_O)-2CH_3_-CH_3_COOH-]^+^; | M2(Hydroxymethyl Armepavine-O-Glucuronide) |
| 2.27 | C_23_H_32_NO_10_ | 518.2034 | 518.2013 | 1.6 | 518[M]^+^;342[M-(GluA-H_2_O)]^+^;192[M-(GluA-H_2_O)-C_9_H_11_0_2_]^+^177[M-(GluA-H_2_O)-C_9_H_11_0_2_-CH_3_]^+^; | M3(Magnoflorine-O-Glucuronide ) |
| 2.16 | C_23_H_30_O_14_ | 529.1565 | 529.1557 | 1.5 | 529[M-H]^-^;355[M-H-C_7_H_10_O_5_]^-^;193[M-H-C_7_H_10_O_5_-Glc]^-^;178[M-H-C_7_H_10_O_5_-Glc-CH_3_]^-^; | 3-({3-[4-(β-Glucopyranosyloxy)-3-methoxyphenyl]-2-propenoyl}oxy)-1,4,5-trihydroxycyclohexanecarboxylic acid |
| 2.01 | C_13_H_16_O_9_ | 315.0732 | 315.0716 | 1.6 | 315[M-H]^-^;153[M-H-Glu]^-^; | 3-Carboxy-4-hydroxy-phenoxy glucoside |
| 2.14 | C_26_H_30_O_12_ | 533.1516 | 533.1515 | 0.2 | 533[M-H]^-^;324[M-H-C_12_H_16_O_3_]^-^;192[M-H-C_14_H_28_O_9_]^-^;178[M-H-C_17_H_22_O_8_]^-^; | Amurensin |
| 2.28 | C_23_H_30_O_14_ | 529.1578 | 529.1557 | 4.0 | 529[M-H]^-^;367[M-H- Glu]^-^;191[M-H- Glu -C_10_H_8_O_3_]^-^;173[M-H- Glu -C_10_H_11_O_4_]^-^; | 3-O-feruloylquinicacid glucoside |
| 2.12 | C_9_H_10_O_5_ | 197.0466 | 197.0450 | 1.6 | 197[M-H]^-^,153[M-H-COO-]^-^; | 2,3,4-Trihydroxy-benzenepropanoic acid |
| 2.4 | C_19_H_28_O_11_ | 431.1564 | 431.1553 | 2.6 | 431[M-H]^-^;299[M-H-C_5_H_8_O_4_]^-^;191[M-H-Glc-C_6_H_6_O]^-^;149[M-H-Glc-C_8_H_8_O]^-^; | 2-(p-hydroxyphenyl) ethanol 1-O-β-D-glucoside |
| 2.4 | C_17_H_19_O_9_ | 369.1160 | 369.1186 | -2.6 | 369[M+H]^+^;193[M+H-C_10_H_8_O_3_] ^+^; | Amurenlactone A/B |
| 2.70 | C_19_H_24_NO_3_ | 314.1749 | 314.1756 | -2.2 | 314[M]^+^;238[M-C_4_H_14_N]^+^;237[M-C_4_H_15_N]^+^;225[M-C_5_H_15_N]^+^;209[M-C_7_H_5_O]^+^;175[M-C_9_H_15_O]^+^; | (-)-Oblongine |
| 2.56 | C_16_H_18_O_9_ | 353.0882 | 353.0873 | 2.5 | 239[M-H-C_4_H_12_N]^-^;224[M-H-C_5_H_14_N]^-^;191[M-H-C_8_H_10_O]^-^;105[M-H-C_13_H_21_NO]^-^; | Chlorogenic acid |
| 2.78 | C_23_H_30_NO_8_ | 448.1997 | 448.1971 | 2.6 | 448[M+H]^+^;286[M+H-Glc]^+^;255[M+H-Glc -CH_2_OH]^+^; | (p-hydroxybenzyl)-6, 7-dihydroxy-N-m ethyltetrahydro iso- quinoline-7-O-p--D-glucopyranoside |
| 3.12 | C_22_H_13_N_2_O_4_ | 369.1169 | 369.1172 | -0.3 | 193[M-H-C_10_H_8_O_3_]; 177[M-H-C_10_H_8_O_4_]; | M4(Reduction of Amurenlactone A/B) |
| 2.85 | C_20_H_24_NO_4_ | 342.1733 | 342.1705 | 2.8 | 406[M+H-OH]^+^;371[M+H-CH_8_O_2_]^+^;303[M+H-C_4_H_8_O_4_]^+^;273[M+H-C_7_H_2_O_4_]^+^; | Phellodendrine |
| 2.83 | C_17_H_22_NO_9_ | 384.1294 | 384.1295 | -0.1 | 384[M]^+^;208[M-(GluA-H_2_O)]^+^;151208[M-(GluA-H_2_O)-CH_2_CO-CH_3_]^+^; | M5(2 × Hydroxylation of Palmatines) |
| 3.01 | C_20_H_24_NO_4_ | 342.1733 | 342.1705 | 2.8 | 327[M-CH_3_]^+^;295[M-C_3_H_11_]^+^;282[M-C_3_H_10_N]^+^;266[M-C_4_H_14_N]^+^;237[M-C_5_H_15_NO]^+^; | Magnoflorine |
| 3.06 | C_19_H_22_NO_4_ | 328.2012 | 314.1756 | -2.9 | 328[M]^+^; | Terahydroreticuline |
| 3.11 | C_27_H_33_NO_10_ | 532.2180 | 532.2183 | -0.3 | 532[M+H]^+^;356[M+H-(GluA-H_2_O)]^+^; | M6(Menisperine-O-Glucuronide) |
| 3.23 | C_16_H_20_O_10_ | 371.0983 | 371.0978 | 1.3 | 371[M-H]^-^; | Syringin |
| 3.15 | C_14_H_14_N_7_O_2_ | 312.1205 | 312.1209 | -1.3 | 312[M+H]^+^;297[M+H-CH_3_]^+^;268[M+H-CH_3_-C_2_H_5_]^+^; | unknowm |
| 3.19 | C_25_H_26_NO_11_ | 500.1554 | 500.1557 | -0.3 | 500[M]^+^;324[M-(GluA-H_2_O)]^+^;309[M-(GluA-H_2_O)-CH_3_]^+^ | M7(Demethyleneberberine-O-Glucuronide ) |
| 3.25 | C_26_H_28_NO_10_ | 514.1708 | 514.1713 | -0.5 | 514[M]^+^;338[M-(GluA-H_2_O)]^+^;323[M-(GluA-H_2_O)-CH_3_]^+^;322[M-(GluA-H_2_O)-CH_4_]^+^; | M8(Jatrorrhizine-O-Glucuronide ) |
| 3.12 | C_17_H_20_O_9_ | 367.1160 | 367.1186 | -2.6 | 367[M-H]^-^;353[M-H-CH_2_]^-^;193[M-H-C_10_H_6_O_3_]^-^;191[M-H-C_10_H_8_O_3_]^-^;189[M-H-C_10_H_10_O_3_]^-^; | 3-O-feruloylquinic acid |
| 3.21 | C_40_H_38_NO_3_ | 581．2232 | 581.2234 | -0.3 | 581[M-H]^-^;419[M-H-Glu]^-^;373[M-H-Glu-C_2_H_5_OH]^-^; | (±)-5,5’-dimethoxylaricire-sinol-4-O-glucoside |
| 3.28 | C_19_H_24_NO_3_ | 314.1749 | 314.1756 | -2.2 | 315[M+H]^+^;271[M+H-C_3_H_8_]^+^;268[M+H-C_3_H_11_]^+^;209[M+H-C_7_H_6_O]^+^;107[M+H-C_12_H_18_NO_2_]^+^; | Lotusine |
| 3.28 | C_17_H_22_O_10_ | 385.1138 | 385.1135 | 0.8 | 209[M-H-(Glu-H_2_O) ]^-^;193[M-H-Glu-OH]^-^;134[M-H-CH_3_-C_8_H_10_O_7_-H_2_O]^-^; | 3-hydroxyl feruloylquinic acid |
| 3.44 | C_21_H_25_NO_4_ | 356.1839 | 356.1862 | -2.3 | 356[M]^+^;325[M-C_2_H_7_]^+^;294[M-C_4_H_14_]^+^;293[M-C_4_H_15_]^+^;232[M-C_7_H_8_O_2_]^+^;192[M-C_10_H_12_O_2_]^+^; | Menisperine |
| 3.6 | C_21_H_22_NO_4_ | 356.1839 | 356.1862 | -2.3 | 356[M]^+^;312[M-C_3_H_7_]^+^;298[M-C_4_H_9_]^+^;296[M-C_4_H_11_]^+^;279[M-C_4_H_12_O]^+^;264[M-C_5_H_15_O]^+^; | Tetrahydropalmatine |
| 3.65 | C_26_H_32_O_8_ | 338.1405 | 338.1406 | -0.3 | 323[M-CH_3_]^+^;322[M –CH_4_]^+^;308[M-2CH_3_]^+^;307[M -2CH_3_-H]^+^;291[M-C_3_H_10_]^+^;290[M-C_3_H_12_]^+^; | Columbamine |
| 3.83 | C_20_H_20_NO_6_ | 370.2016 | 370.2018 | -0.2 | 370[M] ^+^;352[M-H_2_0] ^+^;336[M-H_2_O-CH_3_] ^+^;308[ M-H_2_O -CH_3_-CO]^+^; | M9(Alcohols Dehydration of Palmatine) |
| 3.84 | C_26_H_30_O_7_ | 352.1592 | 352.1594 | -0.6 | 352[M]^+^;336[M-CH_4_]^+^;322[M-CH_3_-CH_3_]^+^;307[M-3CH_3_]^+^;294[M-CH_3_-CO-CH_4_]^+^; | Palmatine |
| 4.05 | C_20_H_18_NO_4_ | 336.1211 | 336.1236 | -2.5 | 336[M]^+^;320[M-CH_4_]^+^;309[M-C_2_H_3_]^+^;306[M-CH_3_-CH_3_]^+^;292[M-CHO_2_]^+^;278[M-C_2_H_3_O_2_]^+^; | Berberine |
| 4.63 | C_26_H_30_O_9_ | 485.1815 | 485.1812 | 0.6 | 485[M-H]^-^;467[M-H-H_2_O]^-^;423[M-H-H_2_O-CO_2_]^-^;411[M-H-H_2_O-C_2_O_2_]^-^; | Rutaevin |
| 4.83 | C_26_H_30_O_8_ | 515.1984 | 515.1984 | 0.0 | 515[M-H+HCOOH]^-^;469[M-H]^-^; | Obaculactone |
| 4.83 | C_26_H_30_O_8_ | 471.1991 | 471.2019 | -5.9 | 471[M+H]^+^;453[M+H-H_2_0]^+^;425[M+H-CO-H_2_O]^+^;339[M+H-C_5_H_8_O_4_^]+^;161[M+H-C_16_H_22_O_6_]^+^; | Obaculactone |
| 6.81 | C_26_H_30_O_7_ | 455.2030 | 455.2070 | -4.0 | 377[M+H-C_4_H_14_O]^+^;331[M+H-C_6_H_4_O_3_]^+^;315[M+H-C_7_H_8_O_3_]^+^;161[M+H-C_17_H_26_O_4_]^+^ | Obacunone |

Note：GluA：Glucuronic acid; Gal: galactose; Glu：Glucose;Glc: β-D-glucose; Rha: α-L-rhamnose;

**Table 7.** Results of potential protein targets analysis based on PharmMapper database

| **PDB ID** | **Protein** | **Fit Score** | **Normalized Fit Score** | **z-score** |
| --- | --- | --- | --- | --- |
| [1P93](http://www.pdb.org/pdb/explore/explore.do?structureId=1nd5) | Glucocorticoid receptor | 4.48 | 0.4978 | 0.952556 |
| 3CP9 | Vascular endothelial growth factor receptor 2 | 3.635 | 0.3635 | 0.34322 |
| 3MDE | Medium-chain specific acyl-CoA dehydrogenase, mitochondrial | 3.957 | 0.6596 | 0.00233406 |
| 1asd | Aspartate aminotransferase | 3.809 | 0.5441 | 0.0349211 |
| 1GS4 | Androgen receptor | 4.634 | 0.7723 | 0.0527787 |
| 2Q9S | Fatty acid-binding protein, adipocyte | 5.467 | 0.6834 | -0.754 |
| 1RBP | Retinol-binding protein | 4.778 | 0.5973 | -0.172517 |
| 1F88 | Rhodopsin | 6.497 | 0.7219 | 1.06604 |
